# Reduced synaptic activity and dysregulated extracellular matrix pathways in midbrain neurons from Parkinson’s disease patients

**DOI:** 10.1038/s41531-022-00366-z

**Published:** 2022-08-10

**Authors:** Shani Stern, Shong Lau, Andreea Manole, Idan Rosh, Menachem Mendel Percia, Ran Ben Ezer, Maxim N. Shokhirev, Fan Qiu, Simon Schafer, Abed AlFatah Mansour, Kile P. Mangan, Tchelet Stern, Polina Ofer, Yam Stern, Ana Paula Diniz Mendes, Jose Djamus, Lynne Randolph Moore, Ritu Nayak, Sapir Havusha Laufer, Aidan Aicher, Amanda Rhee, Thomas L. Wong, Thao Nguyen, Sara B. Linker, Beate Winner, Beatriz C. Freitas, Eugenia Jones, Irit Sagi, Cedric Bardy, Alexis Brice, Juergen Winkler, Maria C. Marchetto, Fred H. Gage

**Affiliations:** 1 Laboratory of Genetics, Salk Institute for Biological Studies, La Jolla, CA USA; 2 Sagol Department of Neurobiology, Faculty of Natural Sciences, University of Haifa, Haifa, Israel; 3 Razavi Newman Integrative Genomics and Bioinformatics Core, Salk Institute for Biological Studies, La Jolla, CA USA; 4 Department of Psychiatry, School of Medicine, Technical University of Munich, Munich, Germany; 5 Department of Medical Neurobiology, Institute for Medical Research Israel-Canada, Faculty of Medicine, Hebrew University of Jerusalem, Jerusalem, Israel; 6 Fujifilm Cellular Dynamics, In, Madison, WI 53711 USA; 7 Department of Immunology and Regenerative Biology, Weizmann Institute of Science, Rehovot, Israel; 8 Department of Stem Cell Biology, University Hospital Erlangen, Friedrich-Alexander-University Erlangen-Nuernberg, Erlangen, Germany; 9 South Australian Health and Medical Research Institute (SAHMRI), Laboratory for Human Neurophysiology and Genetics, Adelaide, SA Australia; 10 Flinders University, Flinders Health and Medical Research Institute (FHMRI), Adelaide, SA Australia; 11 Sorbonne Université, Institut du Cerveau - Paris Brain Institute - ICM, Inserm, CNRS, APHP, F-75013 Paris, France; 12 Department of Molecular Neurology, University Hospital Erlangen, Friedrich-Alexander-University Erlangen- Nürnberg, Nürnberg, Germany; 13 Department of Anthropology, University of California San Diego, 9500 Gilman Drive, La Jolla, CA 92093 USA

**Keywords:** Parkinson's disease, Neurodegeneration

## Abstract

Several mutations that cause Parkinson’s disease (PD) have been identified over the past decade. These account for 15–25% of PD cases; the rest of the cases are considered sporadic. Currently, it is accepted that PD is not a single monolithic disease but rather a constellation of diseases with some common phenotypes. While rodent models exist for some of the PD-causing mutations, research on the sporadic forms of PD is lagging due to a lack of cellular models. In our study, we differentiated PD patient-derived dopaminergic (DA) neurons from the induced pluripotent stem cells (iPSCs) of several PD-causing mutations as well as from sporadic PD patients. Strikingly, we observed a common neurophysiological phenotype: neurons derived from PD patients had a severe reduction in the rate of synaptic currents compared to those derived from healthy controls. While the relationship between mutations in genes such as the *SNCA* and *LRRK2* and a reduction in synaptic transmission has been investigated before, here we show evidence that the pathogenesis of the synapses in neurons is a general phenotype in PD. Analysis of RNA sequencing results displayed changes in gene expression in different synaptic mechanisms as well as other affected pathways such as extracellular matrix-related pathways. Some of these dysregulated pathways are common to all PD patients (monogenic or idiopathic). Our data, therefore, show changes that are central and convergent to PD and suggest a strong involvement of the tetra-partite synapse in PD pathophysiology.

## Introduction

Parkinson’s disease (PD) was first described in 1817 by James Parkinson^1^, who wrote about patients with shaking palsy, i.e., involuntary tremulous motion with lessened muscular power. PD occurs in approximately two of 1000 people and is highly correlated with aging, affecting about 1% of the older population above 60 years of age^2,3^ The main neuropathological symptoms are α-synuclein-containing Lewy bodies and loss of dopaminergic (DA) neurons in the substantia nigra pars compacta. PD patients experience movement difficulties with three cardinal signs: tremor, rigidity, and bradykinesia. Some of the main non-motor symptoms of PD include loss of smell, depression, sleep disorders, and dementia, but a wide range of other symptoms such as excessive saliva^4^ and susceptibility to melanoma^5^ may also be present. With the progression of the disease, Lewy body pathology spreads to many areas of the brain, and Lewy bodies containing α-synuclein aggregates are seen throughout many brain areas such as the hippocampus, hypothalamus, neocortex, and cortex^6–8^.

Approximately 15% of PD cases report familial inheritance of the disease. This percentage may vary significantly in different populations^9^. Hundreds of variants have been observed in several PD genes that showed a clear association, including *α-synuclein* (*SNCA, PARK4*), *parkin* (*PARK2*), *UCH-L1* (*PARK5*), *PINK1* (*PARK6*), *DJ-1* (*PARK7*), *LRRK2* (*PARK8*), *ATP13A2* (*PARK9*), *GBA, VPS35 (PARK17)*, *EIF4G1*, and *PARK16*^10,11^. Thus, PD is not a single disease but a constellation of phenotypes that are displayed variably by patients with different co-morbidities but some commonalities^12^. Some of the genetically characterized forms of the disease are rapidly evolving, with early-onset ages of the mid-30s or mid-40s^13–15^, but some genetic forms have a similar age-onset to idiopathic PD^16^. α-Synuclein immunohistochemistry is currently considered one of the gold standards in the neuropathological evaluation of PD. Aggregates of misfolded α-synuclein in Lewy bodies in DA neurons are now considered a hallmark of PD, but it is not clear whether these Lewy bodies are the cause of neuronal atrophy or a byproduct of the disease^17–19^. In fact, injection of synthetic α-synuclein fibrils into the dorsal striatum of wild-type mice was enough to elicit and transmit disease pathology and neurodegeneration^20^. Similarly, mice carrying mutations that disrupt physiological tetramers of the α-synuclein protein develop brain pathology and neurodegeneration typical of PD^21^. In neurons, α-synuclein physiologically localizes mainly to presynaptic terminals^22–24^. The aggregates of α-synuclein have been observed in cell soma but also in neurites^25^, and they are widespread in various brain areas in PD patients. However, reports show that micro-aggregates are present in the neurites close to presynaptic terminals, causing synaptic impairment sometimes long before the large aggregates that eventually make up the Lewy bodies form^17,26–28^.

Mutations as well as copy number variations in the *SNCA* (*PARK1*) gene, which codes for the α-synuclein protein, have been shown to have a causative effect in PD^13–15,29–35^. α-Synuclein is localized mainly in presynaptic terminals^18^. It helps to maintain the size of the presynaptic vesicular pool as well as vesicle recycling^36,37^, and it functions to help neurotransmitter release, especially dopamine^38–40^. Animal models for mutations and copy number variations in the SNCA gene recapitulate the human motor symptoms and neuronal loss^41–43^. Surprisingly, there is an almost immediate neurophysiological phenotype of a reduction in synaptic activity after the introduction of different SNCA mutations in rodent and human models^37,44–48^. A reduction in synaptic activity has been shown on its own to cause neuronal atrophy in different types of neurons^37,48,49^, suggesting a positive feedback mechanism that further increases neuronal cell death.

Leucine-rich repeat kinase 2 (*LRRK2, PARK8*) is another gene with a causative association with PD^10^. It is the most common form of genetic PD, and mutations are highly prevalent in certain populations^50^. The precise physiological function of *LRRK2* is not completely understood, but recent studies have shown that *LRRK2* is involved in cellular functions such as neurite outgrowth, cytoskeletal maintenance, vesicle trafficking, autophagic protein degradation, and the immune system^51^. Drosophila models with overexpression of *LRRK2* recapitulate PD phenotypes of motor dysfunction and DA cell death^52,53^ and so do C. Elegans overexpression models^54^. However, flies and nematodes do not express a-synuclein and are therefore not a great model for studying PD. Rodent models with *LRRK2* mutations show minimal evidence of neurodegeneration^55,56^ but do show locomotor impairments^56^ and reductions in stimulated dopamine neurotransmission and D2 receptor function. Interestingly, *LRRK2* was found to regulate synaptic vesicle endocytosis and recycling and neurotransmitter release^57–59^. RNA-mediated silencing of *LRRK2* affected postsynaptic currents as well as presynaptic vesicle trafficking and recycling^60^.

Parkin (encoded by *PARK2*) is a ubiquitin-ligase enzyme expressed in the CNS and peripheral tissues^61^. It is a multifunctional protein that is involved in many intracellular processes, and several substrates for it have been identified. Parkin is localized on synaptic vesicles and displays a distribution pattern similar to that of synapsin I, a protein that associates with the cytoplasmic surface of synaptic vesicles^62,63^. Differential fractioning of rat brain lysates revealed that parkin was enriched in the fraction containing PSD-95, a postsynaptic marker^64^. Homozygous and heterozygous mutations are considered risk factors for early-onset PD, though the contribution of the heterozygous form to the onset of PD is still considered controversial^65–67^. Loss of function mutations in the *PARK2* gene cause early and severe degeneration of DA neurons of the substantia nigra pars compacta^68^. Parkin has been shown to negatively regulate the number and strength of excitatory synapses in rats^69^ and to ubiquitinate synaptic proteins^70^. Pathogenic parkin mutations were shown to disrupt glutamatergic synaptic transmission and plasticity by impeding NMDA and AMPA receptor trafficking^71,72^. Mutations in Parkin may therefore cause dysregulation of glutamatergic and DA synapses that eventually culminates in DA neuron cell death.

Overall, quite a few animal model studies suggest that several PD-associated mutations result in a synaptic pathology that occurs before neuronal cell death and may even be the cause of neuronal degeneration^27^. These studies are further supported by other neuroanatomical studies of post-mortem patient brain samples from familial PD cases^73–75^. Here, we used PD patient-derived neurons to study the neuropathology in PD using the induced pluripotent stem cells (iPSCs) technique that has recently been used to study mechanisms of brain disorders^76–80^. Reprogramming adult cells into stem cells is known to erase aging signatures^81,82^; therefore, the neurons derived in this method can be considered young, and even pre-natal, neurons. We found that in all the PD lines [both mutations-derived and sporadic PD (sPD)] there is a significant reduction in the rate of synaptic events in these “young” neurons. The use of iPSCs allowed us to also measure neurons derived from PD patients with no known genetic mutations, who are therefore considered sporadic cases. Our cohort consisted of a few patients with sPD. A few of the patients had mutations in the SNCA gene (one patient with a duplication, one patient with a triplication, and a genetically engineered line with the A53T mutation). One patient had a mutation in the LRRK2 gene, and two patients had a mutation in their Parkin gene. Our results suggest a biological predisposition that exists in PD patients, even without other epigenetic or environmental influences. Furthermore, our methods allowed us to recognize gene expression patterns and gene ontology (GO) terms that are common to neurons derived from patients with different PD mutations as well as to sPD.

## Results

### A drastic reduction in synaptic activity is observed in neurons derived from patients with duplication and triplication of the SNCA gene

**Fig. 1:**
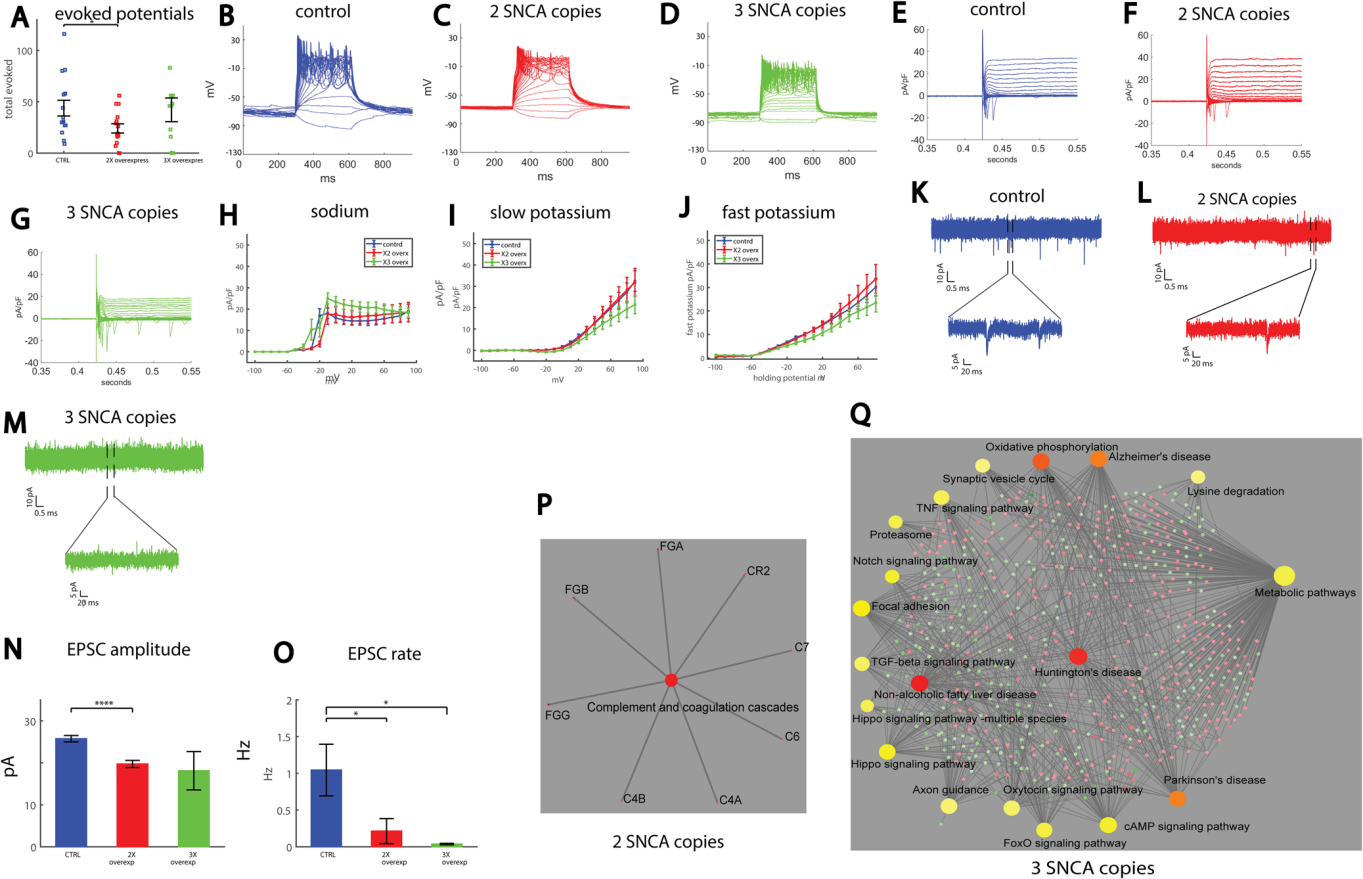
Reduced synaptic activity in neurons of patients with two or three copies of the SNCA gene (denoted as 2X and 3X). **A** Neurons derived from the 2X patient display reduced excitability compared to controls. Representative evoked potential recordings of a control **B**, 2X patient **C**, and 3X patient **D**. Representative traces of sodium and potassium currents in control individuals **E**, 2X patient **F**, and 3X patient **G**. **H** Sodium currents of neurons from 3X patient opened at a less depolarized potential than control DA neurons, whereas those derived from the patient with the double copy opened at a more depolarized potential. **I** Slow potassium currents are reduced in DA neurons derived from 3X patient compared to controls. **J** Fast potassium currents are reduced in DA neurons derived from 3X patient compared to controls. Example traces of recordings of synaptic currents in DA neurons derived from a control individual **K**, the 2X patient **L**, and the 3X patient **M**. The lower image in each is a zoom of the segment denoted by the black bars in the upper image. **N** The mean amplitude of synaptic currents is reduced in DA neurons from 2X patient compared to controls. **O** The average rate of synaptic events is reduced in DA neurons derived from 2X patient and further reduced in 3X patient. **P** Signaling network analysis with the top enriched KEGG pathways for the 2X patient-derived neurons compared to the controls. **Q** Signaling network analysis with the top enriched KEGG pathways for the 3X patient-derived neurons compared to the controls. In this figure and the next figures, asterisks represent statistical significance by the following code: *p value < 0.05, **p value < 0.01, ***p < 0.001, ****p < 0.0001. Error bars represent the standard error in this figure and in the next figures unless stated otherwise.

Our first cohort consisted of a control line (healthy individual, the 40102 line), a PD patient with a duplication of the α- synuclein gene (denoted as 2X), and a PD patient with a triplication of the α-synuclein gene (denoted as 3X). The patient with the α-synuclein triplication had early-onset, autosomal dominant PD at age 38 and was previously described^83^. We differentiated these lines into DA neurons (see “Methods”) and used whole-cell patch-clamp to assess intrinsic properties as well as synaptic activity in the neurons. An immunostaining image for DAPI to mark cell bodies, MAP2 to mark neurons, and tyrosine hydroxylase (TH) to specifically mark dopaminergic neurons is shown in Supplementary Fig. 12P. A total of 15 control neurons, 15 2X neurons, and 15 3X neurons were patch-clamped approximately 50 days after the start of differentiation. The total number of evoked potentials in the 17 first depolarization steps was decreased in the 2X neurons count (see “Methods”, Total evoked action potentials) (*p* = 0.033, Fig. 1A). Representative traces of evoked action potentials are shown in Fig. 1B–D. Next, we used voltage-clamp mode to measure the sodium and potassium currents in the control and PD neurons; representative traces of the currents are shown in Fig. 1E–G. The average sodium currents are presented in Fig. 1H. At −20 mV, control neurons displayed a sodium current of 16.8 ± 3.3 pA/pF, whereas 2X neurons displayed a sodium current of 3.7 ± 1.3 pA/pF (*p* = 0.002 compared to controls), indicating later opening of the sodium channels pf the 2X PD neurons. The slow potassium currents are presented in Fig. 1I. An ANOVA test indicated no significant differences. The fast potassium currents are shown in Fig. 1J. Similarly, running an ANOVA did not indicate significant differences. Analysis of the shape of the action potential is presented in Supplementary Fig. 1A–D.

Recording of Excitatory postsynaptic currents (EPSCs) revealed an altered network activity in both the 2X and 3X neurons. Figure 1K displays a representative recording example of EPSCs in a control neuron, Fig. 1L displays an example recording of EPSCs in a 2X neuron, and Fig. 1M displays an example recording of a 3X neuron. The cumulative distribution of the amplitudes of EPSCs was left-shifted in 2X neurons and further left-shifted in 3X neurons, indicating lower amplitudes of EPSCs (Supplementary Fig. 1E). The average EPSC amplitude was decreased in the 2X neurons (*p* = 0.0006 between the control and 2X neurons, Fig. 1N). The rate of synaptic events per recorded cell is shown in Fig. 1O. The average frequency of EPSCs was 1.04 ± 0.35 Hz for control neurons, 0.21 ± 0.17 Hz for 2X neurons (*p* = 0.04), and 0.037 ± 0.012 Hz for 3X neurons (*p* = 0.01). The cell capacitance was not significantly different between the three groups (Supplementary Fig. 1G). The input conductance was increased in 3X neurons (*p* = 0.0016 between 3X and control neurons, and *p* = 0.0019 between 2X and 3X neurons, Supplementary Fig. 1H). To summarize, a reduction in synaptic activity was observed and exaggerated with α-synuclein gene dosage.

Analyzing the RNA sequencing data and looking at gene ontology and affected KEGG and MsigDB pathways, we found quite a few dysregulated pathways. Four control lines were pooled together for this analysis: 40102, UKERf1JF-X-001, UKERf33Q-X-001, and UKERfO3H-X-001. We plotted the 15 most significant pathways in Supplementary Fig. 13 (up and downregulated). The full lists of GO terms, KEGGS, and MSigDB pathways are presented in Supplementary Tables 3–5. Figure 1P presents the KEGG signaling pathway for RNA extracted from the 2X neurons compared to the control neurons. Similarly, Fig. 1Q presents the top enriched KEGG signaling pathways for RNA extracted from the 3X neurons compared to the control neurons. Interestingly, in the patient with the SNCA triplication, there were many synapse-related dysregulated pathways, with dozens of affected genes (Supplementary Fig. 13D and Supplementary Tables 3, 5), such as synapse part, synaptic vesicle cycle, synaptic transmission, and many more. In the neurons derived from this patient, we also saw a very severe reduction in the rate of synaptic currents, further confirming that synapses were severely disrupted. The full list of the genes involved in the affected pathways is presented in Supplementary Table 6 and consists of both presynaptic and postsynaptic genes. We hypothesize that PD neurons are unable to form or maintain effective synapses, which we detect as a reduction in the rate of synaptic activity with electrophysiology, and this failure, in turn, triggers compensation mechanisms that cause further dysregulation of synapse-related genes. Additionally, in the upregulated pathways, we found “Parkinson’s disease,” Alzheimer’s disease” and “Huntington’s disease” showing commonalities between these neurodegenerative diseases. Several oxidative phosphorylation-related pathways also significantly overlapped with upregulated genes, supporting the known role of oxidative stress in PD, as well as mitochondrial genes that were dysregulated. In the pathways that significantly overlapped with downregulated genes, we found several metabolic pathways and pathways related to protein folding, as well as a few pathways that were not known before to relate to PD and are common in many of our mutation lines as well as sPD (as will be presented in the subsequent figures), such as extracellular matrix pathways (probably the most dysregulated pathways), and PI3K-Akt signaling pathways, lysosome membrane degradation, and focal adhesion. When performing immunocytochemistry (ICC) for two extracellular matrix (ECM) proteins fibronectin and collagen IV, we have observed reduced puncta size in the SNCA triplication neurons compared to the control neurons (see Supplementary Fig. 11).

In the neurons derived from the patient with the SNCA duplication, the “ion transport” pathway and other transporter pathways were dysregulated (See Supplementary Tables 3, 5), which may be related to the changes that we observed in neuronal excitability and ionic currents. In the electrophysiology data, the reduction in synaptic activity was less pronounced than in the patient with the SNCA triplication, further emphasizing the need for electrophysiology for targeting smaller changes that are related to functionality. Several extracellular matrix-related pathways were also dysregulated in the patient with the SNCA duplication, suggesting that there might be changes to the extracellular matrix structure that complement or even precede a possible synaptic degradation. Overall dysregulation of extracellular matrix pathways is common to almost all the PD lines that we worked with.

### A drastic reduction in synaptic activity is observed in DA neurons derived from patients with LRRK2 and Parkin mutations

**Fig. 2:**
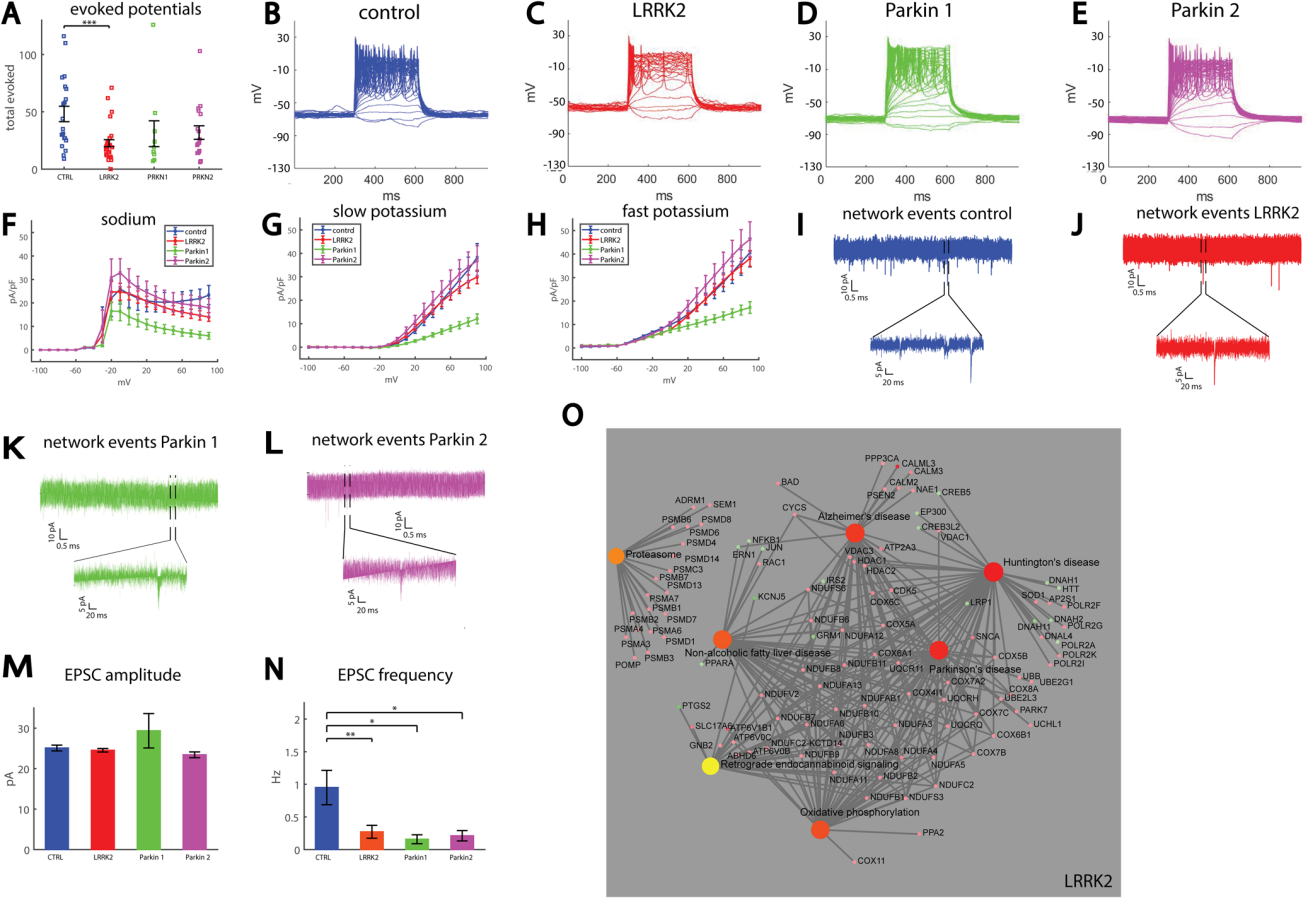
Reduced synaptic activity in neurons derived from patients with LRRK2 and Parkin mutations compared to control dopaminergic neurons. **A** Neurons derived from a patient with a LRRK2 mutation (CHE line) display reduced excitability measured by the total evoked potentials compared to controls. The two patients with the Parkin mutations had a reduction in excitability that was not statistically significant (Parkin1 and Parkin2 patient lines). Representative traces of recordings of evoked potentials in a control neuron **B**, LRRK2 neuron **C**, Parkin1 neuron **D**, and Parkin2 neuron **E**. **F** Sodium currents were reduced in one of the patients with the Parkin mutations (Parkin1) but not in the other patient with Parkin and LRRK2 mutations compared to controls. **G** Slow potassium currents were significantly reduced in neurons derived from the first patient with a Parkin mutation (Parkin1) but not in the second patient with a Parkin mutation (Parkin2) and LRRK2 mutations compared to controls. **H** Fast potassium currents were significantly reduced in neurons derived from the patient with the first Parkin mutation (Parkin1) but not in the second patient with a Parkin mutation (Parkin2) and LRRK2 mutations compared to controls. **I** Example recording of synaptic currents in a neuron derived from a healthy control. **J** Example recording of synaptic currents in a neuron derived from a patient with a LRRK2 mutation (lower plot is a zoom in on the segment denoted in the black segmented lines in the upper graph). **K** Example recording of synaptic currents in a neuron derived from the first patient with a Parkin mutation (Parkin1, lower plot is a zoom in on the segment denoted in the black segmented lines in the upper graph). **L** Example recording of synaptic currents in a neuron derived from the second patient with a Parkin mutation (Parkin2, lower plot is a zoom in on the segment denoted in the black segmented lines in the upper graph). **M** No significant change is observed in the mean amplitude of the neurons with the LRRK2 and Parkin mutations compared to control. **N** The average rate of synaptic events was reduced in neurons derived from a patient with a mutation in the LRRK2 gene and further reduced in patients with a mutation in the Parkin gene. O. Signaling network analysis with the top enriched KEGG pathways for the LRRK2 patient-derived neurons compared to the controls.

Our second cohort consisted of two control (two healthy subjects, 40102 and UKERfO3H-X-001 lines) individuals, one patient with a mutation in the LRRK2 and two patients with mutations in the Parkin gene. We differentiated these lines using a DA human neuronal protocol^84^ (see “Methods”) and used whole-cell patch-clamp to assess intrinsic properties as well as synaptic activity in the neurons (*n* = 20 control neurons, *n* = 31 from the patient with the LRRK2 mutation, *n* = 12 from one patient with a Parkin mutation and *n* = 15 neurons from the second patient with a Parkin mutation, for a total of 27 neurons recorded for the Parkin mutation). The total number of evoked potentials in the 17 first depolarization steps was decreased in the LRRK2 DA neurons (see “Methods”, Total evoked action potentials) and in the neurons with the Parkin mutation (*p* = 0.0005 between control and LRRK2 mutation, *p* = 0.059 between control and the two Parkin mutations, pooling the data of the two mutations together, Fig. 2A). Representative traces of evoked action potentials are shown in Fig. 2B–E. Next, we measured the sodium and potassium currents in voltage-clamp mode in the control, LRRK2, and Parkin DA neurons. The average sodium currents are presented in Fig. 2F. The average slow potassium currents are presented in Fig. 2G and the average fast potassium currents are presented in Fig. 2H. The sodium, slow, and fast potassium currents were reduced in one of the Parkin lines (PET) (using an ANOVA, *p* = 0.0002 for sodium currents, *p* = 0.02 for slow potassium currents, and *p* = 0.01 for fast potassium currents). To assess network activity, we next measured EPSCs. Representative traces of measured EPSCs are shown in Fig. 2I (control), 2J (LRRK2 mutation), 2K (first patient with a Parkin mutation termed Parkin1), and 2L (the second patient with a Parkin mutation, termed Parkin2). There was no significant change in the average amplitude of the synaptic currents (Fig. 2M). There was a significant reduction in the frequency of synaptic currents in all three PD-causing mutations. Control neurons had 0.95 ± 0.25 Hz, LRRK2 0.27 ± 0.1 Hz, Parkin1 0.16 ± 0.07 and Parkin2 0.21 ± 0.08 Hz (LRRK2 compared to control *p* = 0.009, Parkin1 compared to control *p* = 0.027, Parkin2 compared to control *p* = 0.019, Fig. 2N). When we performed imaging of calcium transients (see Supplementary Fig. 10), we observed smaller correlations between the spontaneously active neurons of the DA neurons with the Parkin mutation compared to the control neurons, suggesting that neurons with the Parkin mutation are less connected, further supporting the data of the patch-clamp showing a reduction in the EPSC frequency.

We also analyzed several features of the shape of the spikes. There was a significant decrease in the spike amplitude in the LRRK2 mutation (LRRK2 vs. control, *p* = 0.01; Parkin pooled together vs. control, *p* = 0.07, Supplementary Fig. 2A). There were no significant changes in the spike threshold (Supplementary Fig. 2B), the spike width (Supplementary Fig. 2C), or the fast afterhyperpolarization (AHP) amplitude (Supplementary Fig. 2D). The cumulative distribution of the amplitude of EPSCs was similar between the control and the LRRK2 and Parkin lines (Supplementary Fig. 2E). There were no significant differences in the capacitance of the neurons (Supplementary Fig. 2F), and the input conductance was significantly larger in the first line with the Parkin mutation compared to the control (*p* = 0.03, Supplementary Fig. 2G).

The top 10 GO terms and KEGG and MSigDB over-representation analyses for the DA neurons with the LRRK2 and Parkin mutations are presented in Supplementary Fig. 14, and the full list is shown in Supplementary Tables 3, 5. The signaling network analysis with the top enriched KEGG pathways is presented in Fig. 2O for the LRRK2 mutant neurons compared to the control neurons. The differentially expressed gene list is presented in Supplementary Table 4. Interestingly, several synaptic transmission pathways were significantly overlapped with upregulated genes in the LRRK2 mutation, in agreement with our electrophysiological data, with approximately 10–20 affected genes in each of these pathways; these included “regulation of synaptic transmission”, “excitatory synapse” and more (these are shown in Supplementary Tables 3, 5). Similar to the neurons derived from patients with the SNCA copy number variation, Parkinson’s disease, Alzheimer’s disease, and Huntington’s disease, oxidative phosphorylation, and oxidation reduction-related pathways were significantly overrepresented among upregulated genes. In addition, mitochondrial pathways were overrepresented among upregulated genes, presenting more evidence that mitochondrial defects are present in PD. Among the terms significantly overlapping with downregulated genes we found many affected pathways that were related to the extracellular matrix, PI3K-Akt signaling pathway, lysine degradation, focal adhesion, and FoxO signaling pathways, as well as protein folding-related pathways. When performing ICC for two ECM proteins fibronectin and collagen IV, we have observed reduced puncta size in the LRRK2 neurons compared to the control neurons (see Supplementary Fig. 11). Some of these pathways were not previously known to be associated with PD, but they were dysregulated in most of our PD lines, showing new possible cellular defects that may be targeted for treatment. For the DA neurons derived from the PD patient with the Parkin mutation, there were not many significantly dysregulated pathways. These did include “transport vesicle membrane.”

### A reduction in synaptic activity is observed in neurons derived from sPD patients

**Fig. 3:**
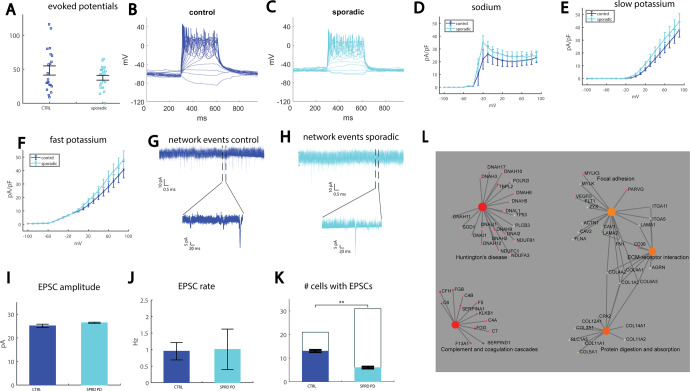
A reduction in the number of neurons with synaptic activity is observed in neurons derived from a sPD patient. **A** No significant change was observed in the excitability measured by the total evoked potentials in the neurons derived from the sPD patient compared to controls. **B** Representative example of evoked action potentials in a control neuron. **C** Representative example of evoked action potentials in a neuron derived from a sPD patient. **D** No significant changes were observed in the sodium currents. **E** No significant changes were observed in the slow potassium currents. **F** No significant changes were observed in the fast potassium currents. **G** Example recording of synaptic currents in a neuron derived from a healthy control (the lower plot presents a zoom in on the segment denoted in the black dashed lines in the upper graph). **H** Example recording of synaptic currents in a neuron derived from a patient with sPD (the lower plot presents a zoom in on the segment denoted in the black dashed lines in the upper graph). **I** No significant change was observed in the mean amplitude of the synaptic currents in the sPD neurons. **J** No significant change was observed in the mean rate of the synaptic currents in the sPD neurons. **K** A significant reduction in the number of neurons that had synaptic activity was observed in sPD neurons compared to controls. **L** Signaling network analysis with the top enriched KEGG pathways for the sPD patient-derived neurons compared to the controls.

We continued the study by differentiating neurons from a PD patient with no PD-causing mutations (UKERfAY6-X-001). We recorded *n* = 20 control neurons (two patients: 40102 and UKERfO3H-X-001) and *n* = 33 neurons from the sPD patient. There was no significant change in the total evoked action potentials (Fig. 3A for averages and 3B, 3C for representative traces). The average of the sodium currents is presented in Fig. 3D. The average over the slow potassium currents is presented in Fig. 3E and the average of the fast potassium currents is presented in Fig. 3F. There was no significant difference between the sPD and the control neurons in any of the sodium, slow potassium, and fast potassium currents. Representative EPSC traces are presented in Fig. 3G, H. There were no significant differences in the mean amplitude (Fig. 3I) or the mean rate (Fig. 3J) of EPSCs. However, when counting the number of neurons that had synaptic activity (see “Methods”), we did see a significant reduction in this number in the sPD neurons (*p* = 0.0013, Fig. 3K). Furthermore, analysis of the decay time constant of the synaptic events revealed a faster decay in sPD neurons, with an average of 2.3 ± 0.1 ms for control neurons and 1.5 ± 0.07 ms for sPD neurons (*p* = 9e−12, the distribution is presented in Supplementary Fig. 3). Spike shape analysis did not reveal any significant changes in the spike height (Supplementary Fig. 4A). The spike was significantly narrower in the sPD neurons (control 6 ± 0.8 ms, sPD 3.6 ± 0.2 ms, *p* = 0.0035, Supplementary Fig. 4B). The spike threshold was significantly more negative in the sPD neurons (control −18.4 ± 1.3 mV, sPD −24.6 ± 1 mV, *p* = 0.0006, Supplementary Fig. 4C). There was no significant change in the fast AHP (Supplementary Fig. 4D) or the capacitance (Supplementary Fig. 4F), and the input conductance was larger in sPD neurons (control 0.44 ± 0.12 nS, sPD 0.89 ± 0.16 nS, *p* = 0.05, Supplementary Fig. 4G). The cumulative distribution of amplitudes of EPSCs was similar between the control and sPD neurons (Supplementary Fig. 4E).

Three sPD lines (UKERfRJO-X-001, UKERfAY6-X-001, and UKERfM89-X-001) were pooled together for the analysis of gene expression, plus four control (lines 40102, UKERf1JF-X-001, UKERf33Q-X-001, and UKERfO3H-X-001). The top 10 GO terms, KEGG, and MSigDB dysregulated pathways for the DA neurons derived from sPD patients are presented in Supplementary Fig. 15, and the full list is shown in Supplementary Tables 3–5. The signaling network analysis with the top enriched KEGG pathways for the sPD neurons compared to the control neurons is presented in Fig. 3L. There were a few dysregulated pathways that were synapse-related such as regulation of synaptic structure, regulation of synaptic organization, regulation of synaptic plasticity, and more. The most dysregulated pathways were related to nasopharyngeal carcinoma, with almost 250 affected genes and a false discovery rate (FDR) of 1.6e–105. Other highly dysregulated pathways were related to the cilium. Like the other genetic PD lines, the extracellular matrix was highly affected, and more dysregulated pathways that were repeatedly affected in our PD lines were focal adhesion, collagen processes, PI3K-Akt, protein digestion and absorption, and pathways related to reactive oxygen species and metabolic processes. When performing ICC for two ECM-proteins fibronectin and collagen IV, we have observed reduced puncta size in the sPD neurons compared to the control neurons (see Supplementary Fig. 11). Several hypoxia-related pathways were affected as well.

### A reduction in synaptic activity is observed in neurons derived from more sPD patients using a different protocol and selecting a subset of the neurons

We next analyzed data that were acquired using a different differentiation protocol (see “Methods”, second DA differentiation); in addition, only neurons that were defined as type 5 neurons (see definition in “Methods”) were used for the analysis. Strikingly, despite the different methods used both in differentiating the neurons and in analyzing the data, the main phenotype of a reduction in synaptic activity was present in the sPD neurons as well.

**Fig. 4:**
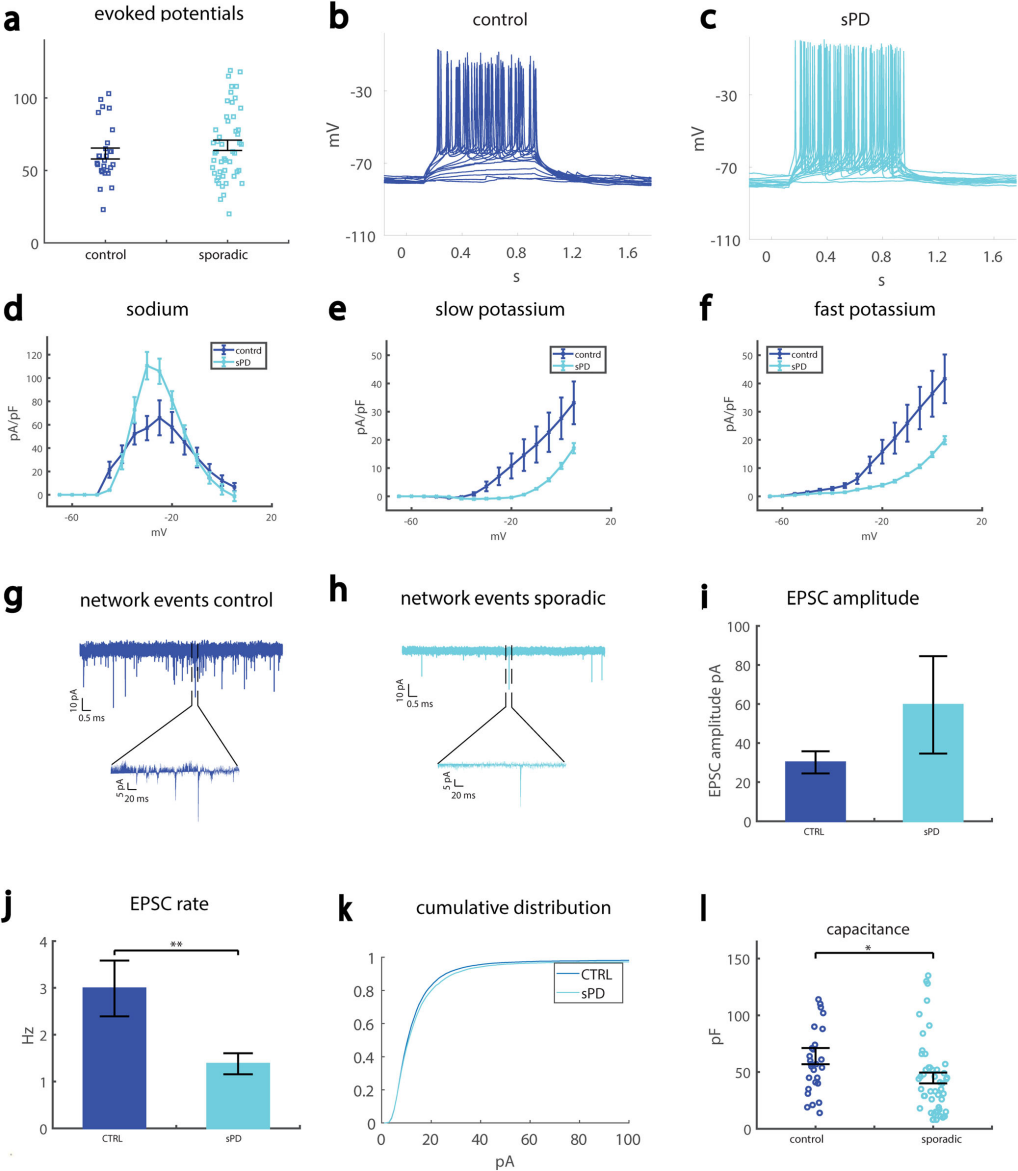
Reduced synaptic activity in neurons derived from two sPD patients compared to control neurons. **A** No significant change was observed in the excitability measured by the total evoked potentials in the neurons derived from the sPD patient compared to controls. **B** Representative example of evoked action potentials in a control neuron. **C** Representative example of evoked action potentials in a neuron derived from a sPD patient. **D** Sodium currents are increased in the sPD neurons compared to the controls. **E** The slow potassium currents are reduced in the sPD neurons compared to the control neurons. **F** The fast potassium currents are reduced in the sPD neurons compared to the control neurons. **G** A representative trace of synaptic currents in a control neuron (the lower plot presents a zoom-in on the segment denoted in the black dashed lines in the upper graph). **H** A representative trace of synaptic currents in an sPD neuron (the lower plot presents a zoom in on the segment denoted in the black dashed lines in the upper graph). **I** The mean amplitude of the synaptic currents was increased, but not significantly, in the sPD neurons. **J** The mean rate of synaptic currents was significantly reduced in the sPD neurons. **K** The cumulative distribution of the sPD neurons is slightly right-shifted, indicating large amplitudes of synaptic currents. **L** The capacitance of sPD neurons was significantly reduced in sPD neurons compared to control neurons.

We recorded from control individual (line 40102) and two sPD patients (UKERfRJO-X-001, UKERfR66-X-001). It should be noted that one of the patients had a heterozygous missense mutation in the EIF4G1 gene (UKERfRJO-X-001). The number of total evoked action potentials was not different between control and sPD neurons (averages presented in Fig. 4A and representative traces are presented in Fig. 4B, C). The averages of the sodium currents are presented in Fig. 4D, the averages of the slow potassium currents are presented in Fig. 4E, and the averages of the fast potassium currents are presented in Fig. 4F. Representative traces of synaptic activity are presented in Fig. 4G (control) and 4H (sPD). The average amplitude of the synaptic currents was increased in sPD neurons, but not significantly (Fig. 4I). The average rate of synaptic currents was significantly reduced in the sPD neurons (control 3 ± 0.6 Hz, sPD 1.4 ± 0.2 Hz, *p* = 0.0037, Fig. 4J). The cumulative distribution of the amplitude of synaptic currents was slightly right-shifted in the sPD neurons. The capacitance was reduced in the sPD neurons, indicating smaller cells (control 64 ± 38 pF, sPD 45 ± 33 pF, *p* = 0.02). Performing action potential shape analysis, we did not observe any significant changes in the spike height (Supplementary Fig. 5A) or the spike width (Supplementary Fig. 5B). The amplitude of the fast AHP was larger in the sPD neurons (control −5.7 ± 0.8 mV, sPD −10.9 ± 0.6 mV, *p* = 0.0000035, Supplementary Fig. 5C), and the threshold for evoking an action potential was more depolarized (control −43 ± 0.8 mV, sPD −39.9 ± 0.6 mV, *p* = 0.0016, Supplementary Fig. 5D).

### A reduction in synaptic activity is observed in neurons derived from an edited iPSC line with an inserted A53T mutation in the SNCA gene

**Fig. 5:**
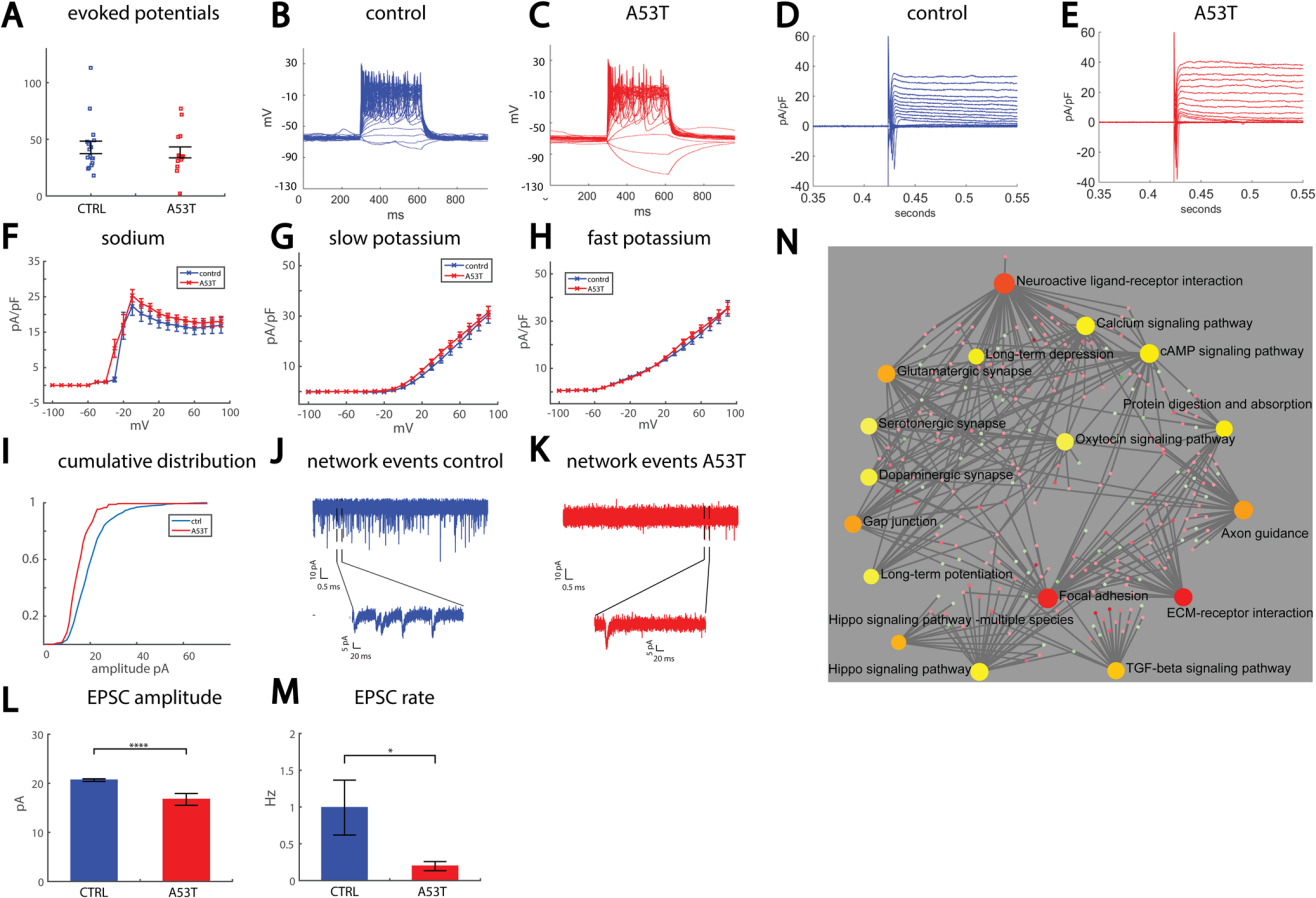
Reduced synaptic activity in neurons derived from an engineered line of iPSCs with an A53T mutation in the SNCA gene compared to isogenic controls. **A** No significant change in the excitability of the A53T neurons compared to control neurons. **B** Example recording of action potentials in current-clamp mode of a control neuron. **C** Example recording of action potentials in current-clamp mode of an A53T neuron. Sodium currents of A53T neurons open at a lower depolarization potential than control neurons. **D** Example recording of sodium and potassium currents in voltage-clamp mode in a control neuron. **E** Example recording of sodium and potassium currents in voltage-clamp mode in an A53T neuron. **F** No significant changes were observed in the sodium currents of the A53T neurons compared to controls. **G** No significant changes were observed in the slow potassium currents in the A53T neurons. **H** No significant changes were observed in the fast potassium currents in the A53T neurons. **I** The cumulative distribution of the amplitudes of EPSCs is left-shifted in A53T neurons compared to control neurons, indicating lower amplitudes in the A53T neurons. **J** Representative trace of the synaptic currents in a control neuron (the lower plot presents a zoom-in on the segment denoted in the black dashed lines in the upper graph). **K** Representative trace of the synaptic currents in an A53T neuron (the lower plot presents a zoom-in on the segment denoted in the black dashed lines in the upper graph). **L** The average amplitude of synaptic currents was significantly reduced in A53T neurons compared to control neurons. **M** The average rate of synaptic currents was significantly reduced in A53T neurons compared to the control neurons. **N** Signaling network analysis with the top enriched KEGG pathways for the A53T mutant compared to the controls.

We next performed experiments on neurons derived from a healthy human subject whose fibroblasts were reprogrammed into iPSCs and on an engineered line, in which the A53T mutation was edited into the SNCA gene (isogenic lines). The healthy line and the edited mutated line were differentiated into DA neurons by a differentiation technique that is proprietary to Fujifilm Cellular Dynamics Inc (CDI) (iCell DopaNeurons^85^). Having an edited healthy line allowed us to observe a similar genetic background, thereby measuring the neuronal changes that occurred specifically due to the A53T mutation. iCell DopaNeurons (CDI) were previously shown to have a protein expression pattern that supported a midbrain lineage DA phenotype^85^. Using whole-cell patch-clamp, we measured the functional features of A53T neurons compared to the control neurons. Fourteen A53T and 17 control neurons were recorded. The total number of evoked potentials in 17 depolarization steps (see “Methods”) was not significantly different between A53T neurons (39 ± 5) and control neurons (43 ± 6) (Fig. 5A and representative traces in Fig. 5B, C). Using voltage clamping we measured the sodium/potassium currents in the neurons (representative traces are shown in Fig. 5D, E). Sodium currents were not significantly different, except for the opening of the sodium channels; at −30 mV, control neurons displayed a sodium current of 1.6 ± 0.7 pA/pF, whereas A53T neurons displayed a sodium current of 10.4 ± 2.5 pA/pF (*p* = 0.0012, Fig. 5F). Slow and fast potassium currents were not significantly different between A53T neurons and controls (Fig. 5G, H). Spike parameters were not altered in the A53T neurons (Supplementary Fig. 6A–D). The cumulative distribution curve of the amplitude of synaptic currents was left-shifted in A53T compared to controls, indicating lower amplitudes of synaptic currents (Fig. 5I). Representative example recordings of EPSCs for control neurons (Fig. 5J) and an A53T neuron (Fig. 5K) are shown. The mean amplitude of synaptic currents was significantly reduced in A53T neurons (16.7 ± 1.2 pA) compared to control neurons (20.6 ± 0.3 pA, *p* = 2e−6, Fig. 5L). The mean rate of synaptic events was significantly reduced in A53T neurons (0.2 ± 0.06 Hz) compared to control neurons (1 ± 0.4 Hz, Fig. 5M, *p* = 0.05). Cell capacitance was smaller, but not significantly, in A53T neurons (20.2 ± 1.6 pF) compared to control neurons (23 ± 1.4 pF, Supplementary Fig. 6E). The input conductance was decreased, but not significantly, in A53T neurons (Supplementary Fig. 6F). Further imaging of neurons stained for TH showed that the A53T neurons were smaller and had fewer, less arborized neurites (Supplementary Fig. 7A–H). We similarly analyzed immunostaining images for the other PD lines and did not find changes in neurites’ lengths (see Supplementary Fig. 8). The mean percentage of neurons with neurite beading was 3.7 ± 0.6% in control cultures and 28 ± 1.8% in A53T cultures (*p* = 2e−10, Supplementary Fig. 7I–K). Increased expression of the SNCA gene was observed in the A53T mutant neurons, and also in the LRRK2 mutant neurons, and the neurons derived from the patient with the triplication in the SNCA gene (Supplementary Fig. 9).

The top 10 affected KEGG and MsigDB pathways and GO terms are presented in Supplementary Fig. 16, and the full list is given in Supplementary Tables 3–5. The signaling network analysis with the top enriched KEGG pathways of the A53T mutant neurons compared to the controls is presented in Fig. 5N. There were many dysregulated synapse-related pathways such as dopaminergic synapse, postsynaptic membrane, presynaptic membrane, chemical synaptic transmission, and more. Similar to the other PD lines presented in this study, there were many extracellular matrix-related affected pathways as well as pathways related to focal and cell adhesion, collagen processes, protein digestion and absorption, and PI3K-Akt signaling pathways. It is interesting to note that similar findings of synaptic defects, both at the electrophysiological and morphological level, as well as dysregulation of pre- and post-synaptic factors and transsynaptic adhesion molecules identified by transcriptome analysis, were previously reported^44^.

### Protein aggregates and altered morphology in the A53T SNCA mutated iDopaNeurons compared to control neurons

**Fig. 6:**
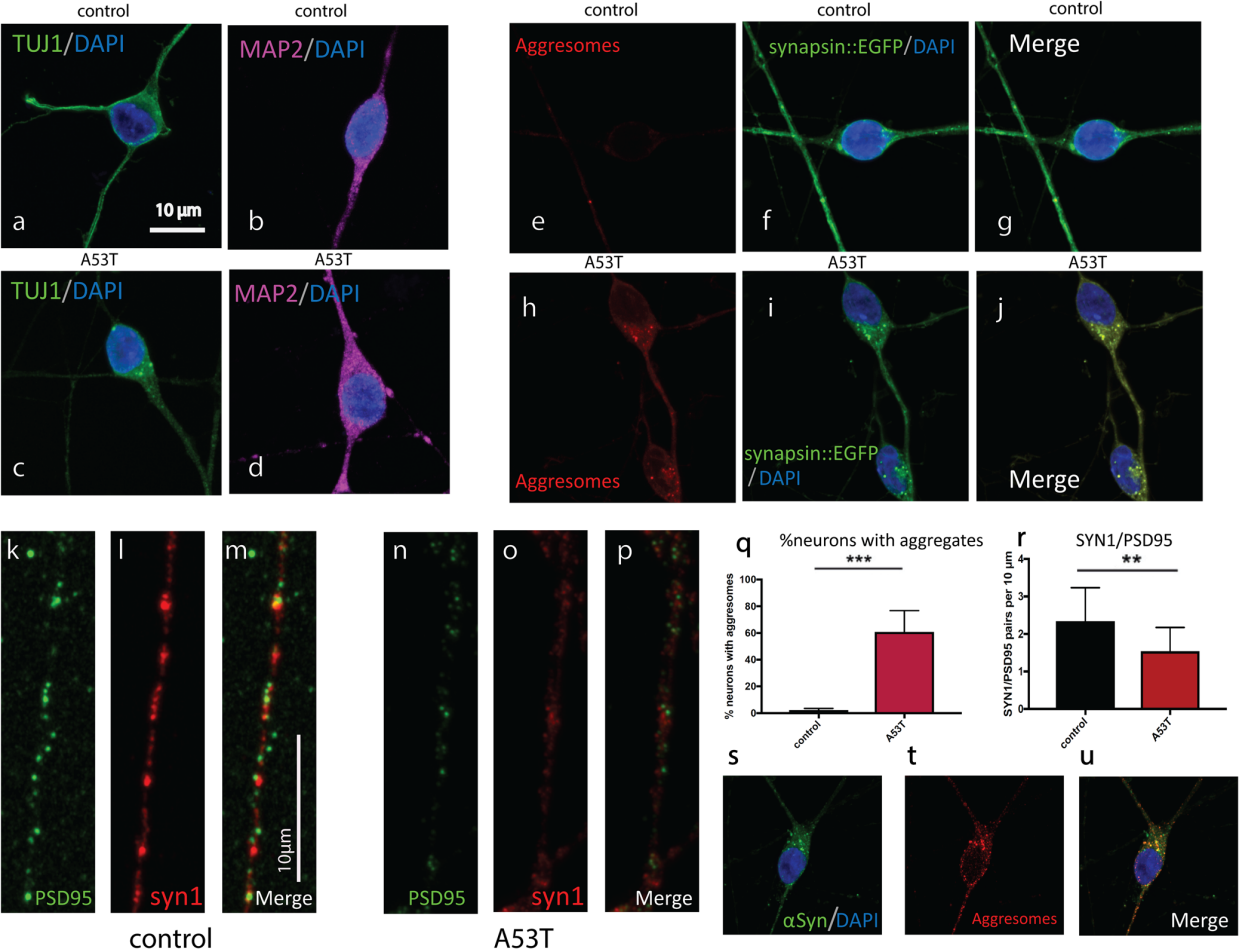
Protein aggregates in A53T dopaminergic neurons. **a** Representative image showing expression of neuron-specific class III beta-tubulin/ 4′,6-diamidino-2-phenylindole (TUJ1/DAPI) in control dopaminergic neurons. Scale bar 10 µm. **b** MAP2/DAPI expression in control dopaminergic neurons. Similar representative images of A53T dopaminergic neurons expressing TUJI/DAPI (**c**) and MAP2/DAPI **d**. **e**–**g** Control dopaminergic neurons exhibit almost no protein aggregates, as can be seen in the aggresomes and merged image with synapsin::EGFP/DAPI on the right. **h**–**j** A53T dopaminergic neurons display high levels of protein aggregates that is observed in the aggresomes staining. **k**–**p** Immunostaining for postsynaptic density protein 95 (PSD95 in green) and synapsin1 (syn1) reveals a drastic decrease in the ratio of synapsin1 to PSD95, indicating fewer synapses. **q** Quantification of protein aggregates shows a drastic increase in the number of neurons harboring protein aggregates in the A53T mutated neurons compared to controls. **r** Quantification of the ratio of syn1/PSD95 shows a drastic and significant decrease in A53T dopaminergic neurons compared to controls. **s**–**u** α-Synuclein staining and aggresomes reveal co-localization of some of the protein aggregated with α-synuclein. Error bars represent standard deviations in this figure.

We next delved deeper to look for additional morphological and cellular alterations that occurred in the engineered A53T iDopaNeurons neurons compared to controls. The neurons were stained for neuronal markers TUJ1 and MAP2, and both A53T and control neurons expressed these markers (Fig. 6a–d). Misfolded protein aggregates were observed in A53T neurons but rarely seen in control neurons (Fig. 6e–j). The number of aggregates was quantified using the aggresomal kit and imaging in ultra-high resolution, 60.8 ± 7.1% of the A53T neurons displayed protein aggregates (*n* = 99), compared to 2.2 ± 0.6% of the control neurons (*n* = 96, *p* < 0.0001, Fig. 6q). To assess the number of synapses, we co-stained for synapsin1 and the post-synaptic marker PSD95. The number of puncta pairs Syn1/PSD95 density was significantly reduced in A53T cultures; 2.3 ± 0.2 pairs in 10 µm were observed in control cultures, whereas only 1.5 ± 0.16 (*n* = 15 neurites) pairs in 10 µm were observed in A53T cultures (*p* = 0.009, Fig. 6k–p, r). To assess how many of the misfolded protein aggregates were α-synuclein positive, we co-stained α-synuclein/aggresomes in the A53T neurons. Some of the aggregates contained α-synuclein protein (Fig. 6s–u).

### Seeking commonly affected pathways in PD neurons

**Fig. 7:**
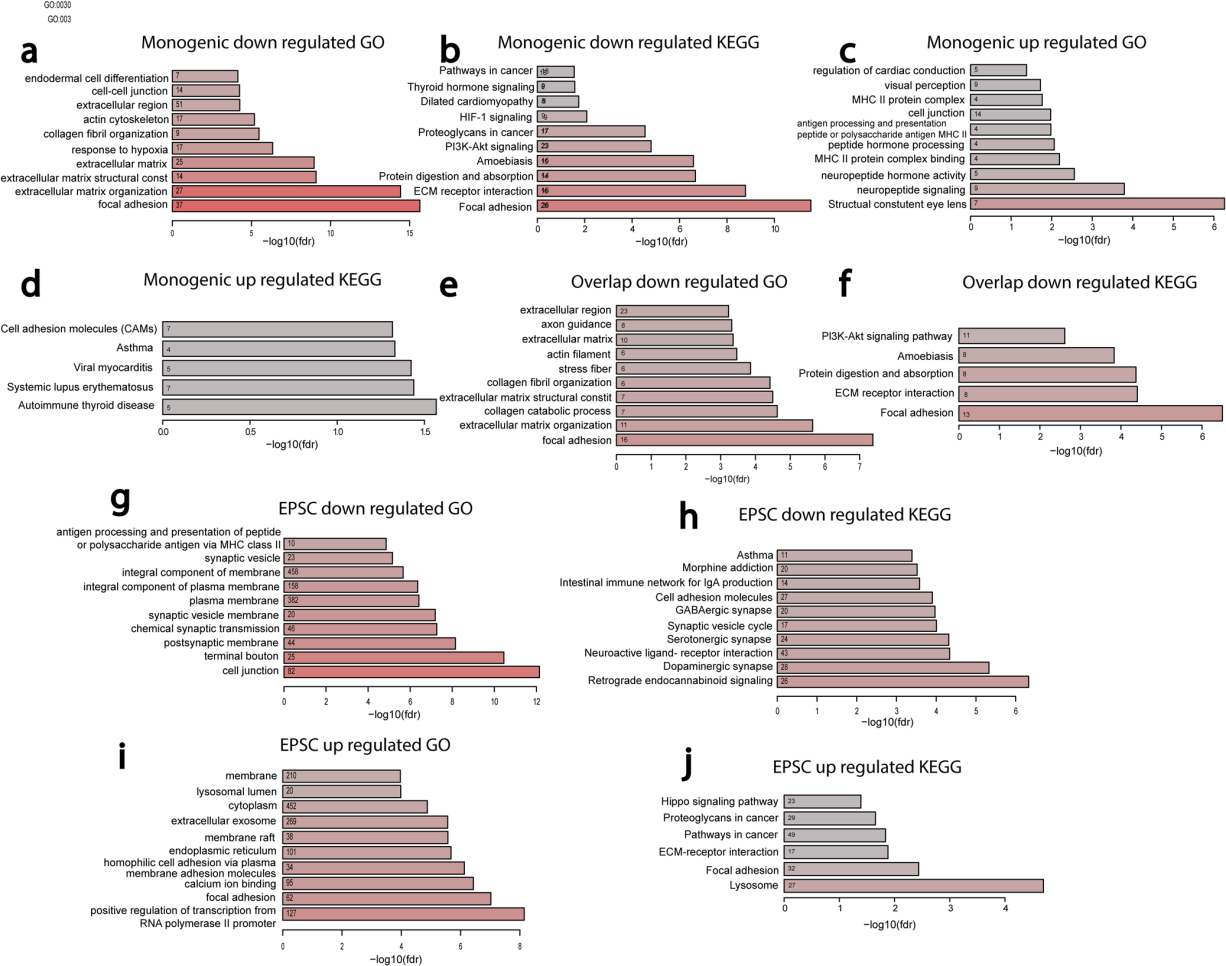
Pathways affected in monogenic (samples of all mutations pooled together) PD and pathways that are affected in neurons with a low rate of EPSCs. **a** Top downregulated GO terms in the monogenic PD compared to controls. **b** Top downregulated KEGG pathways in monogenic PD compared to controls. **c** Top up-regulated GO terms in the monogenic PD compared to controls. **d** Top up-regulated KEGG pathways in monogenic PD compared to controls. GO terms for upregulated genes in the monogenic PD compared to controls. **e** Commonly down-regulated GO terms in the monogenic neurons and the sPD neurons compared to the control neurons. **f** Commonly downregulated KEGG pathways in the monogenic neurons and the sPD neurons compared to the control neurons. **g** Downregulated GO terms in DA neurons that have a low rate of EPSCs. **h** Downregulated KEGG pathways in DA neurons that have a low rate of EPSCs. **i** Upregulated GO terms in DA neurons that have a low rate of EPSCs. **j** Upregulated KEGG pathways in DA neurons that have a low rate of EPSCs.

PD patients share similar symptoms despite completely different genes causing the disease, or even when there is no defined genetic cause. Therefore, we sought to find common pathways between neurons derived from PD patients with different mutations. We started by pooling the RNA sequencing results from all our lines with PD-causing mutations—SNCA duplication, SNCA triplication, LRRK2, and Parkin—and we looked for differential expression compared to the four control lines. The 10 top terms enriched among up and down-regulated genes are presented in Fig. 7a–d (GO and KEGG pathways). The full list, as well as MsigDB dysregulated pathways, are given in Supplementary Tables 3–5. A clear picture emerges of pathways that were strongly affected in neurons derived from all PD lines with mutations; some of them were not previously known to be associated with PD. Collagen-related pathways and the extracellular matrix were very strongly affected in the PD lines, as was confirmed by ICC experiments at the protein level for Fibronectin and collagen IV (Supplementary Fig. 11). Focal adhesion was another pathway that repeated throughout the monogenic PD lines, as well as the PI3K-Akt signaling pathway, pathways related to cancer and oxidoreductase activity, and protein digestion and absorption. Importantly, synapse-related pathways such as synapse, synaptic membrane, postsynapse, and more were commonly dysregulated in our monogenic PD lines. In the up-regulated pathways, we found cell adhesion molecules (CAM), which are molecules that interact with the extracellular matrix and may be a compensation mechanism for the reduced collagen and other extracellular matrix-related genes. We also looked for overrepresented GO terms for genes that were common in the monogenic PD lines and the sPD lines and these are presented in Fig. 7e, f. The extracellular matrix is commonly implicated in both monogenic and sPD. Other dysregulated pathways were collagen pathways, focal and cell adhesion, PI3K-Akt signaling pathway, protein digestion and absorption, and a few pathways related to hypoxia. Pathways related to oxidative stress such as reactive oxygen species and oxidative stress were also dysregulated. Age-related pathways such as brain up, Alzheimer’s disease up, and cellular senescence were also commonly dysregulated in both monogenic and our sPD lines. Pathways that might be related to synapse function that were dysregulated included cell-cell junction, axon guidance, cell junction assembly, vasculature development, and regulation of neuron development projection.

### Seeking pathways related directly to a reduction in synaptic transmission rate

The common electrophysiological phenotype that we observed in all PD lines was a drastic and significant reduction in the rate of synaptic current events. Therefore, we were interested to determine the affected pathways when the differential expression was taken relative to the EPSC rate of the investigated cell line (see “Methods”). The 10 top upregulated pathways for neurons with reduced EPSC rates are presented in Fig. 7g–j and the entire list of dysregulated GO terms and KEGG and MsigDB pathways are presented in Supplementary Tables 3–5. As expected, many synaptic pathways were dysregulated when comparing neurons that had a high-frequency rate of synaptic activity vs. neurons with a low rate of synaptic events. These included, for example, dopaminergic synapse, glutamatergic synapse, GABAergic synapse, serotonergic synapse, aminergic neurotransmitter loading into synaptic vesicles, filopodium, axon guidance, and many more. Other dysregulated pathways were similar to those that were dysregulated in PD, probably since PD lines exhibited a reduced synaptic event rate. These included many extracellular matrix pathways, focal adhesion, CAMs, melanosome, lysosome, cilium, mitochondrial pathways, endoplasmic reticulum-related pathways, and several cancer-related pathways.

## Discussion

PD affects the lives of nearly one million people in the US and is the most prevalent movement disorder. The hallmarks of PD are aggregates of the α-synuclein protein that are more specific to areas in the brain with a high density of DA neurons. In some PD cases, Lewy bodies appear in those DA-dense areas. These Lewy bodies are composed of protein aggregates whose main component is the α-synuclein protein. However, it is not clear if the Lewy bodies are causing the neuronal cell death that is observed in high-density DA neuron areas or is a side effect of other processes that occur and are the actual triggers for neurodegeneration. Recent studies suggest that degeneration starts as micro aggregates of the α-synuclein protein form, long before Lewy bodies appear. Strikingly, there are several different PD-causing mutations, but most patients do not have a known genetic origin and are considered idiopathic or sporadic. Finding phenotypes that are common to PD early in the lifetime of neurons (and the patients) will help to understand disease mechanisms and develop an effective treatment. Furthermore, the use of patient-derived neurons helps to mitigate the problem of the lack of animal models for sporadic PD.

Here we report a neurophysiological phenotype that is common to DA neurons derived from PD patients for the following mutations: the A53T SNCA mutation, SNCA copy number variations (duplication as well as triplication), LRRK2, Parkin, and importantly, sPD patients. Using whole-cell patch-clamp, we found that neurons derived from PD patients all exhibited a significant reduction in the rate of synaptic activity. It is known that neurons that are plated sparsely have a low survival rate^86,87^; neurons need interactions with their neighboring neurons for survival. Therefore, the low connectivity that we observed in the PD neurons may exacerbate neuronal death caused by different mechanisms. Several labs have shown that reprogramming of adult cells into iPSCs erases aging signatures and epigenetic modifications^81,82^, and therefore the neurons in our cultures are considered to be young, and even pre-natal neurons. Consequently, the observed phenotype of a reduction in synaptic activity in these very young neurons indicates an early process and a biological predisposition that is present in the patients’ DA neurons, probably long before patients exhibit any motor deficits. Similar findings were described in SNCA mutations in mice^42,43,45,47,48^. In these mice, the earliest phenotype that was observed was a reduction in synaptic activity, followed by protein aggregates that, after weeks and sometimes months, evolved into the loss and death of DA neurons and motor dysfunction. Overall, our results imply that neuronal cultures derived from human patients using iPSC techniques are a good model for studying PD progression, starting with this prodromal phenotype in the neurons. Moreover, this phenotype may have important implications for the early diagnosis and prediction of disease onset.

The α-synuclein gene plays a major role in PD. The SNCA gene was only dysregulated in the neurons derived from the patients with the LRRK2, A53T, and SNCA triplication mutations, with a very large increase in the neurons derived from the patient with the SNCA triplication (Supplementary Fig. 9). It is interesting to note that the neurons derived from the patient with the SNCA duplication do not significantly overexpress SNCA, perhaps due to compensation mechanisms. However, we revealed shared dysregulated pathways analyzing gene expression of neurons derived from PD patients with different genetic mutations as well as the sporadic form of the disease and this was often consistent with, and supportive of the electrophysiology. Among these dysregulated pathways and genes, we found the CAMs. Several CAM families have been shown to localize at the synapses^88,89^ and to influence the assembly and function of synapses in the CNS. The PI3K-Akt signaling pathways were also overrepresented among downregulated genes in both monogenic and sPD, and they have been shown to recruit PSD-95 to synapses^90^. The FoxO pathway, another commonly overrepresented pathway among downregulated genes in our PD lines, has also been shown to play an important role in synaptic growth, synaptic vesicle exocytosis^91^, and the promotion of synaptic plasticity^92^. The latter two pathways have been shown to interact^93^. Proteoglycans and collagen fibers also exhibited dysregulation in both our monogenic and sPD lines. These make up the extracellular matrix, which more and more evidence suggests is a part of the tetrapartite synapse. The extracellular matrix-related pathways were strongly dysregulated in almost all our PD lines. Overall, we saw the dysregulation of many genes and pathways that affected synaptic formation. We hypothesize that this gene expression dosage dysregulation prevents neurons derived from PD patients from establishing effective synapses, measured in our electrophysiological experiments as a reduced rate of synaptic events. We hypothesize that PD neurons have a reduced ability to form operative synapses, which reduces the rate of synaptic events and triggers homeostatic mechanisms that take place; a vast dysregulation of genes associated with synaptic transmission-related pathways then occurs, as we observed when analyzing gene expression in our PD lines. It is interesting to note that, in those PD lines where there was a stronger decrease in the synaptic event’s rate, we also observed more abundant synaptic-related pathways that were overrepresented among upregulated genes. In the LRRK2 and the SNCA duplication, where the reduction in the synaptic rate was not as severe, we detected fewer synaptic pathways that were significantly enriched among upregulated genes, further stressing the importance of electrophysiology for the detection of subtler changes. Overall, our results strongly suggest that PD pathology starts at the synapse (as our in vitro neurons are young, and therefore reflect early brain events), in agreement with previous reports and hypotheses regarding the role of disrupted synapses in PD^27,94,95^. Studies have shown that loss of synaptic terminals exceeds the loss of DA cell bodies^74^ and that α-synuclein aggregates at the presynaptic terminals before forming Lewy bodies^18,28^; these results are supported by our findings of young and rejuvenated DA neurons that already exhibit synaptic deficits.

To summarize, our work demonstrates an early phenotype that is common to neurons from several PD mutations as well as sPD patients. It also demonstrates that there are common pathways that are affected in and common to PD patients (mutation-driven or sporadic). Importantly, these findings reveal that PD can be studied using iPSC-derived neuron technology, allowing us to trace the disease progression step by step. The affected pathways that we have identified through the analysis of gene expression should now be considered important targets for further research.

## Methods

A written informed consent was provided by all the participants in the study to take part in the study.

### Ethics

The study was approved by the Salk institute with the following approvals: IRB 09-0003 and ESCRO 07-009.

### Human patients

Supplementary Tables 1 and 2 (see Supplementary) present the clinical features of the human patients who participated in this study. The first cohort, listed in Supplementary Table 1, was diagnosed by Dr. Juergen Winkler, and the second cohort (Supplementary Table 2) was diagnosed by Dr. Alexis Brice. A written informed consent was provided by all the participants in the study to take part in the study.

### Dopaminergic (DA) neurons

DA neurons were generated from iPSCs based on a previously described protocol^84^ with modifications^96^. This protocol robustly produces over 80% DA neurons within the neuronal population in the culture. We performed an assessment of the neuronal types in our different PD lines and controls, and the protocols yields a high percentage of DA neurons with almost no glutamatergic neurons and approximately 5–10% GABAergic neurons (see Supplementary Fig. 12). In brief, iPSCs were dissociated into a single cell suspension with TrypLE and replated on Matrigel-coated plates at a density of 40,000 cells/cm^2^ in mTesR medium (Stem Cell Technologies). Cells were allowed to propagate with a daily change of medium for two days. Two days after the propagation of the cells, the differentiation process was started by switching into KSR medium (DMEM F-12 with Glutamax, 15% KO-SR, 1% NEAA, 1% Antibiotic-Antimycotic, 0.1 mM b-mercaptoethanol); this day was regarded as day 0. The medium was gradually changed to N2 medium (DMEM F-12 with Glutamax, 1% N2 supplement, 1% Antibiotic-Antimycotic) from day 5 to day 10 (day 5 and 6: 75% KSR : 25% N2; day 7 and 8: 50% KSR : 50% N2; day 9 and 10: 25% KSR : 75% N2). From Day 11 onward, the medium was switched to B27 medium (Neurobasal medium, 2% B27 supplement, 1% glutamax, 1% Antibiotic-Antimycotic, 10 ng/mL BDNF, 10 ng/mL GDNF, 1 ng/mL TGF3, 0.2 mM ascorbic acid and 0.1 mM cAMP). Cocktails of small molecules were added to the culture throughout the differentiation process (10 µM SB431542 on day 0–4; 100 nM LDN-193189 on day 0–12; 2 µM purmorphamine, 0.25 µM SAG, 100 ng/mL FGF8 on day 1–6; 3 µM CHIR99021 on day 3–12). Cells were replated onto Matrigel-coated coverslips on day 20 and further matured in B27 medium until day 30. On day 30, the base medium was switched to BrainPhys medium^97^ until, on day 45–50, whole-cell patch-clamp recording was performed. All neurons that had a good quality patch-clamp seal were included in the analysis. The neurons in one of the experiments (results presented in Fig. 4) were grown and differentiated at the Bardy lab, using a published protocol^97,98^. This protocol produces a smaller proportion of tyrosine hydroxylase (TH)-positive neurons. Furthermore, the analysis was performed only on neurons that were classified as “type 5” neurons by this protocol.

### Plating neurons from a commercial control line and an affected line

We utilized commercial neurons from Fujifilm Cellular Dynamics International (CDI), which are a more homogeneous culture of neurons and produce more than 80% pure midbrain DA neurons The detailed nomenclatures/catalog numbers for the cells used in this paper are iCell DopaNeurons, 01279, catalog number: C1028; and MyCelliDopaNeurons, C1279, catalog number: C1113, genotype: SNCA (A53T alfa synuclein mutation). The cells were defrosted according to the protocol on the User’s Guide from CDI and counted. The medium used was Brainphys (Stem Cell Technologies), with the addition of supplements, according to the manufacturer’s instructions: iCell Neural Supplement B (catalog number: M1029) and iCell Nervous System Supplement (catalog number: M1031), N2 supplement, Laminin, and antibiotics (penicillin/streptomycin). For patch clamp electrophysiology experiments, we used Poly-L-ornithine- and Laminin-coated, 24-well plates with coverslips in each well, and we plated 4–6 × 10^5^ cells per well. Recordings started after a week in culture. For the RNA sequencing experiments, we plated 1 × 10^6^ cells per well on six-well plates. For immunostaining, we plated about 1 × 10^5^ cells per well of 8 chamber ibidi slides.

### iCell DopaNeuron cultures on multi-electrode arrays (MEAs)

Control iCell DopaNeurons (Fujifilm Cellular Dynamics Inc.) and MyCell *SNCA* A53T DopaNeurons were thawed and dotted (~80k cells) onto a 48-well MEA plate (Axion Biosystems) using the iCell DopaNeuron MEA application protocol. Cultures were treated with BrainPhys medium (Stem Cell Technologies), with 50% medium changes performed every two to three days for 20 days. Five-minute recordings were made on DIV20 post-plating (*N* = 24). Raw voltage recordings were processed via a Butterworth (200–4 kHz) filter; action potentials were then detected by a 5.5 standard deviation detection threshold, and network-level bursting behaviors were analyzed off-line using the Axion’s NeuralMetric toolbox via the ‘Envelope’ algorithm (threshold factor 3, minimum inter-burst-interval 10 s, burst inclusion percentage 75, minimum number of electrodes percentage 25) and synchrony index (20 ms)^48^. The activity and network-level bursting behaviors assessed included mean firing rate, network bursting rate (bursts per minute: BPM), intensity (Hz), and duration (seconds). A two-tailed student t-test was used to assess statistical differences on each measure independently between the two groups of neurons.

### Electrophysiology—Whole-cell patch-clamp

Neurons on glass coverslips were transferred to a recording chamber in a standard recording medium containing (in mM) 10 HEPES, 4 KCl, 2 CaCl_2_, 1 MgCl_2_, 139 NaCl, 10 D-glucose (310 mOsm, pH 7.4). Whole-cell patch-clamp recordings were performed on days 9–12 days after plating for A53T vs. iDOPA CDI lines and for 40 days after plating for the other DA protocols. Patch electrodes were filled with internal solutions containing (in mM) 130 K-gluconate, 6 KCl, 4 NaCl, 10 Na-HEPES, 0.2 K-EGTA, 0.3 GTP, 2 Mg-ATP, 0.2 cAMP, 10 D-glucose, 0.15% biocytin and 0.06% rhodamine. The pH and osmolarity of the internal solution were brought close to physiological conditions (pH 7.3, 290–300 mOsmol). Signals were amplified with a Multiclamp700B amplifier and recorded with Clampex 10.2 software (Axon Instruments). Data were acquired at a sampling rate of 20 kHz and analyzed using Clampfit-10 and the software package Matlab (2018b, The MathWorks Inc., Natick, MA, 2000). All measurements were conducted at room temperature. For post-synaptic current measurements, 40 µM of bicuculline was applied and cells were held at −60 mV when currents were recorded (−70 mV after correction of junction liquid potential).

### Analysis electrophysiology

#### Total evoked action potentials

Cells were typically held in current-clamp mode near −60 mV with a steady holding current, and current injections were given starting 12 pA below the steady holding current in 3 pA steps 400 ms in duration. A total of 35 depolarization steps were injected in current-clamp mode. Neurons that needed more than 50 pA to be held at −60 mV were discarded from the analysis. The total number of action potentials was counted in the first 17 depolarization steps.

#### Action potential shape analysis

The first evoked action potential was used for spike shape analysis (with the lowest injected current needed for eliciting an action potential). Spike threshold was the membrane potential at which the slope of the depolarizing membrane potential increased drastically, resulting in an action potential (the first maximum in the second derivative of the voltage vs. time). The fast (5 ms) AHP amplitude was calculated as the difference between the threshold of the action potential and the value of the membrane potential 5 ms after the potential returned to cross the threshold value at the end of the action potential. The spike amplitude was calculated as the difference between the maximum membrane potential during a spike and the threshold. Action potential width was calculated as the time it took for the membrane potential to reach half the spike amplitude in the rising part of the spike to the descending part of the spike (Full Width at Half Maximum).

#### Input conductance

The input conductance was calculated around the resting membrane potential by measuring the current with the cell held in voltage-clamp mode first at −70 mV and then at −50 mV. The difference in currents divided by the difference in membrane potential (of 20 mV) is the calculated input conductance.

#### Sodium and potassium currents

The sodium and potassium currents were acquired in voltage-clamp mode. Cells were held at −60 mV, and voltage steps of 400 ms were made in the range of −90 mV to 80 mV. Currents were normalized by the cell capacitance.

#### Fast and slow potassium currents

We measured the fast potassium current by the maximum current immediately following a depolarization step, typically within a time window of a few milliseconds. The slow potassium currents were obtained at the end of the 400 ms depolarization step.

#### Capacitance

The capacitance was measured by the membrane test of the Clampex SW.

#### Synaptic activity

Excitatory synaptic activity was measured in voltage-clamp mode with 40 μM bicuculline applied in the recording medium. The neurons were held at −60 mV and currents were measured in the patched neuron. We measured both the amplitude of these currents and their rates. A neuron was defined as having synaptic activity if it had more than 20 events in a 60 s recording and as not having synaptic activity if it had fewer than 20 events in 60 s.

### RNA-Sequencing analysis

Sequenced reads were quality-tested using FASTQC^99^ v0.11.5 and aligned to the hg19^100^ human genome using the STAR aligner^101^ version 2.5.3a. Mapping was carried out using default parameters, filtering non-canonical introns and allowing up to 10 mismatches per read, and only keeping uniquely mapped reads. The genome index was constructed using the gene annotation supplied with the hg19 Illumina iGenomes collection^102^ and sjdbOverhang value of 100. Raw or TPM (Transcripts per million) gene expression was quantified across all gene exons with HOMER^103^ using the top-expressed isoform as a proxy for gene expression, and differential gene expression was carried out on the raw counts using the edgeR^104^ package version 3.28.1. For each disease type, differentially expressed genes were defined as having a false discovery rate (FDR) <0.05 when comparing two experimental conditions. We also separated monogenic subjects from sPD and compared each group separately to controls using the exact test function in edgeR. We then combined all subjects (monogenic, sPD, and control) and treated the EPSC rate as a continuous variable; we conducted differential expression analysis using the GLM model in edgeR. In both cases, differentially expressed genes were defined as having an FDR < 0.05. A GO enrichment test and KEGG pathway analysis were performed using the program DAVID Bioinformatics Resources 6.8^105^. Overrepresentation of GO terms and KEGG pathway was determined by FDR < 0.05. MsigDB^106^ overrepresentation analysis was carried out using HOMER findGO.pl using the corrected Benjamini & Yakutieli method for multiple testing correction^107^.

### Immunocytochemistry and cell imaging

Neuronal cultures were fixed with 4% paraformaldehyde for 15 min at room temperature and then treated with PBS containing 0.1% Triton X-100. After a 15 min PBS wash, cells were blocked with 5% BSA in PBS for 1 h, then incubated with the primary antibody in PBS at 4 °C overnight and the next day after a few PBS washes with secondary antibodies for 1 hour at ambient temperature. This process was followed by a 10 min incubation with DAPI and a final set of PBS washes. The coverslips were mounted on glass slides using PVA-DABCO. Primary antibodies used were rabbit anti-Tuj1, (1:500, Covance), chicken anti-MAP2 (1:400, Abcam), rabbit anti-αSynuclein (1:500, Invitrogen), rabbit anti-TH (1:500, Pel-Freez), mouse anti-PSD95 (1:500, Life Tech), rabbit anti-Synapsin I (1:500, Calbiochem), anti-Fibronectin (1:200, Sigma). Corresponding Alexa Fluor^TM^ secondary antibodies were then used (1:1000). For detecting protein aggregates, the PROTEOSTAT® Aggresome Detection kit was used according to the manufacturer’s instructions.

Confocal z-stacks were acquired with a Zeiss LSM 880 Airy scan microscope (Carl Zeiss, Microimaging Inc.) using 405 nm Diode laser, 488 nm Argon, and 543 nm HeNe lasers with a Plan NeoFluar 40×/1.3 oil DIC or a Plan-Apochromat 63×/1.4 oil DIC objective.

### Imaging of cell morphology

Neurons stained for TH were imaged and analyzed with Neurolucida SW (MBF Bioscience), where neurites and soma were manually traced.

### Calcium imaging

To image calcium transient neuronal cultures were incubated for 60 min in the recording solution (128 mM NaCl, 4 mM KCl, 1 mM CaCl_2_, 1 mM MgCl_2_, 45 mM sucrose, 10 mM glucose, and 10 mM HEPES; pH is titrated to 7.4) in the presence of 4 μg/ml cell-permeant Fluo5-AM (Abcam). Cultures were then placed in fresh recording solution and imaged. The calcium transients were imaged at 10 Hz similar to previous experiments described^108^.

### Statistical analysis

Unless otherwise stated, *p* values were calculated using a two-sample t-test (two-tailed).

## Supplementary information

Supplementary

Supplementary Table 3

Supplementary Table 4

Supplementary Table 5

Supplementary Table 6

## Data Availability

The datasets generated and analyzed during the current study are available in the figshare repository in the following links: https://figshare.com/articles/dataset/ephys_data_7z/14635452, https://figshare.com/articles/dataset/MsigDB_xlsx/19665885, https://figshare.com/articles/dataset/DEGs_xlsx/19665897. The patients information is provided in https://github.com/Precision-Disease-Modeling-Lab/NPJ-Parkinson-Disease-NPJPARKD-00785R1. The RNA sequencing raw data is available in GEO Series record GSE207533.

## Code availability

The electrophysiological and Statistical analysis were performed using Matlab (2018b, The MathWorks Inc., Natick, MA, 2000). The Matlab scripts used for the analysis are available from the corresponding author upon reasonable request.
