# Supplementary material for: Reduced synaptic activity and dysregulated extracellular matrix pathways in midbrain neurons from Parkinson’s disease patients
Source: NPJ Parkinsons Dis. 2022 Aug 10;8:103. doi: 10.1038/s41531-022-00366-z (PMC9365794; doi:10.1038/s41531-022-00366-z)
Supplement: Supplementary file 1 — Supplementary [file 41531_2022_366_MOESM1_ESM.pdf]

Supplementary figures and legends

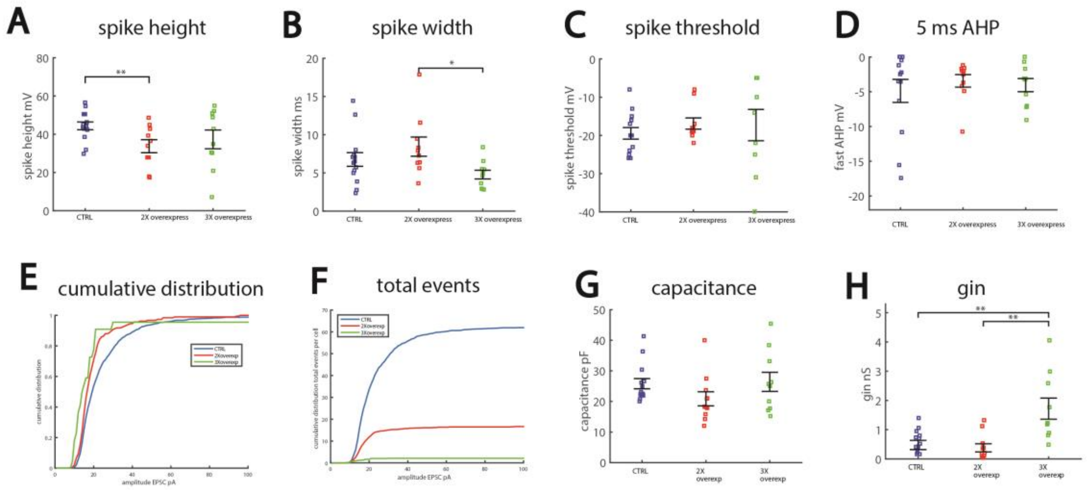

Supplementary Figure 1. A. Spike height is decreased in dopaminergic neurons derived from patients with a double copy of the SNCA gene compared to controls. B. Spike width is broadened in dopaminergic neurons derived from patients with a double copy (2X) of the SNCA gene compared to patients with a triple copy (3X) of the SNCA gene. C-D. No changes in the threshold for evoking an action potential and in the fast after-hyperpolarization (AHP) were observed between the 2X and 3X patient’s neurons and the controls. E. The cumulative distribution of the amplitude of synaptic currents shows that dopaminergic neurons derived from 2X and 3X patients have a significantly smaller amplitude of synaptic currents than control dopaminergic neurons. F. The total number of events per recorded cell is drastically reduced in dopaminergic neurons derived from 2X and even more in the 3X patients compared to controls. G. The capacitance is reduced (but not significantly) in the neurons derived from the 2X patient. H. The input conductance (gin) is increased in dopaminergic neurons derived from the 3X patients compared to controls and 2X patients. * p value<0.05, **p value<0.01. Error bars represent the standard error in this figure.

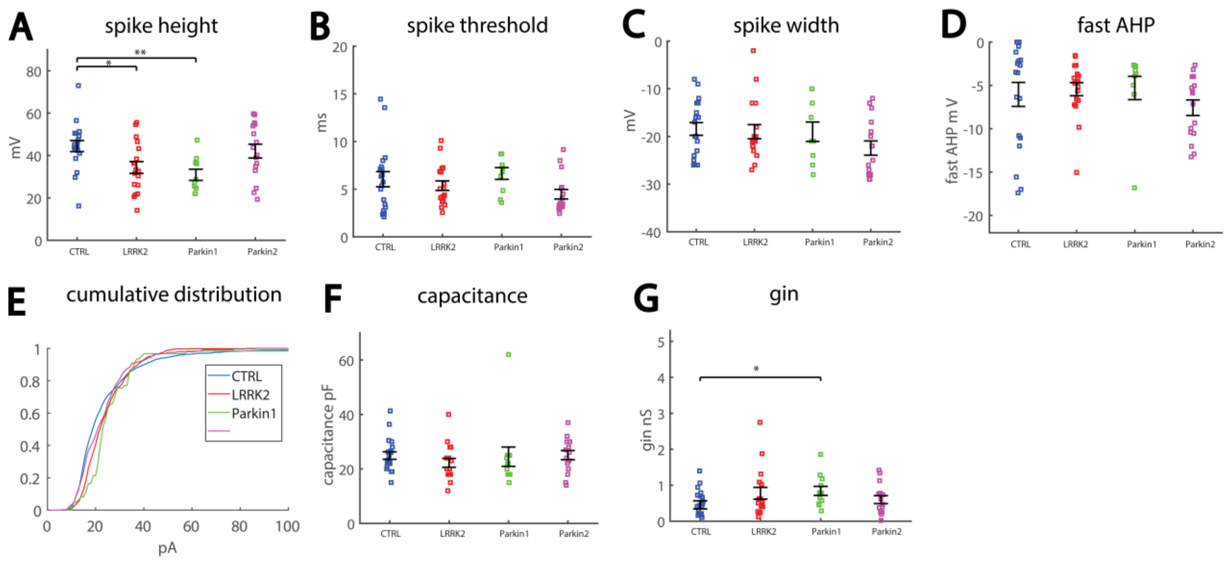

Supplementary Figure 2. A. Spike height was significantly smaller in dopaminergic neurons derived from the patient with the first Parkin mutation and the LRRK2 mutation, but not in the second patient with the second Parkin mutation compared to controls. B. Spike threshold was not significantly different between the lines. C. Spike width was unchanged between the lines. D. There was no change in the fast after-hyperpolarization (AHP) observed between the patient's neurons and the controls. E. The cumulative distribution of the amplitude of EPSCs shows that there is no change to the distribution of amplitudes of EPSCs between control dopaminergic neurons and the ones derived from the patient with mutations in the LRRK2 and Parkin genes. F. No change was observed in the capacitance between neurons derived from healthy controls and neurons derived from the Parkin and LRRK2 mutations carrying patients. G. A significant increase was observed in the input conductance (gin) of dopaminergic neurons derived from one of the first patient with the Parkin mutation. * p value<0.05, **p value<0.01. Error bars represent the standard error in this figure.

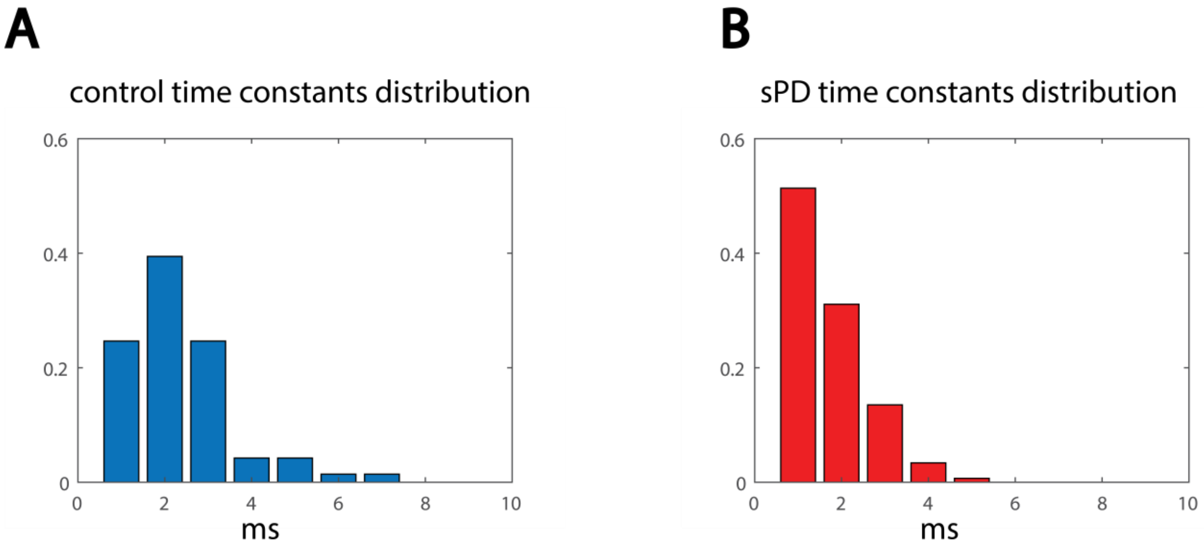

Supplementary Figure 3. The distribution of the decay time constants in control and sPD neurons indicates shorter decay time in sPD neurons. A. The distribution in control neurons. B. The distribution in sPD neurons.

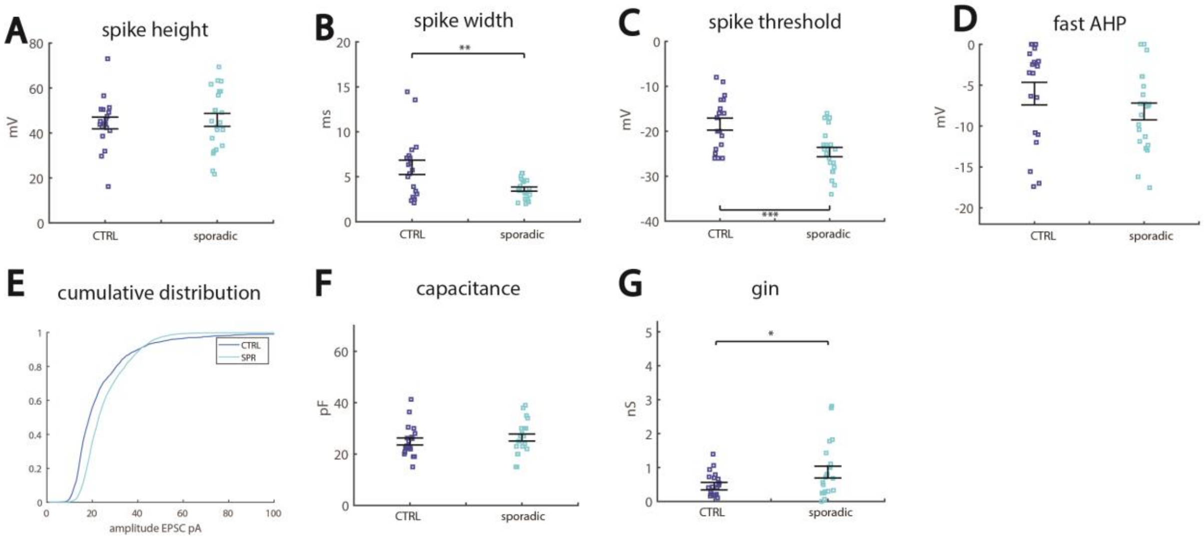

Supplementary Figure 4. A. No significant changes were observed in the spike height between control and sPD patient. B. Spike width was significantly narrower in neurons derived from the sPD patient compared to those derived from the healthy control. C. Spike threshold was significantly less depolarized in the neurons derived from the sPD patient compared to the healthy control. D. There was no change in the fast after-hyperpolarization (AHP) observed between the patient's neurons and the controls. E. The cumulative distribution of the amplitude of synaptic currents shows that there is no change in the distribution of amplitudes of synaptic currents between control and sPD dopaminergic neurons. F. No significant change was observed in the capacitance of the control and sPD neurons. G. A significant increase

is observed in the input conductance (gin) of sPD dopaminergic neurons and controls. * p value<0.05, **p value<0.01. Error bars represent standard error in this figure.

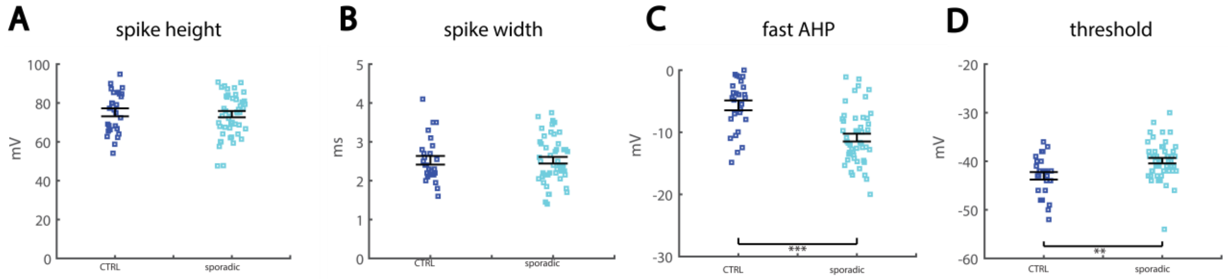

Supplementary Figure 5. A. No significant changes were observed in the spike height between control and sPD patient. B. No significant changes were observed in the spike width between control and sPD patient. C. Increased amplitude of the fast AHP was observed in the neurons derived from the sPD patient compared to the controls. D. A more depolarized threshold was observed in neurons derived from the sPD patient compared to the controls. **p value<0.01, ***p<0.001. Error bars represent the standard error in this figure.

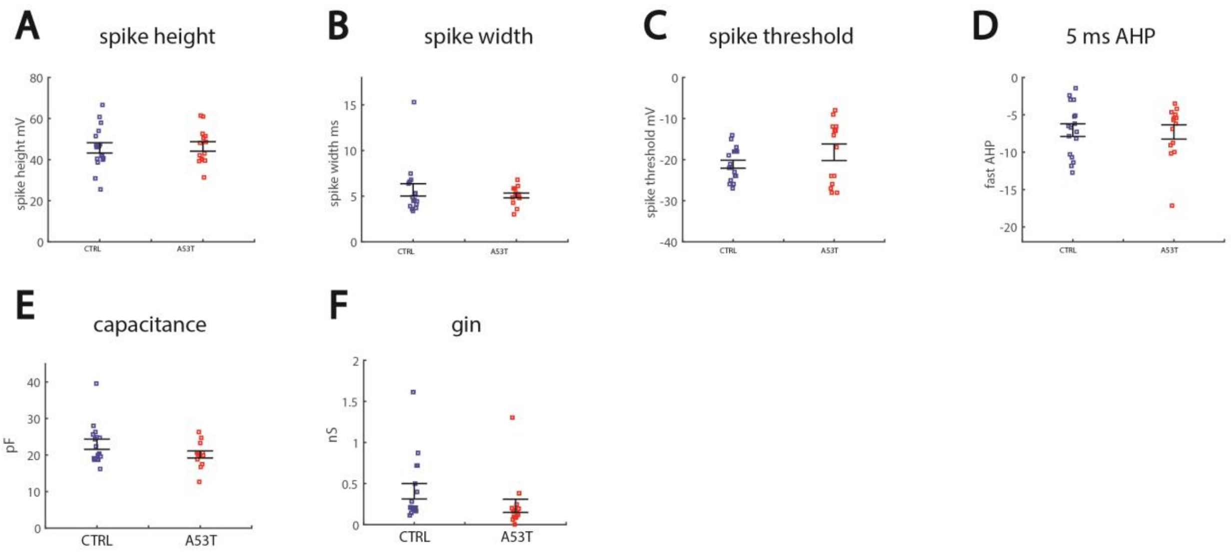

Supplementary Figure 6. A-F. No significant changes were observed in the spike amplitude (A), spike width (B), spike threshold (C), the fast AHP (D), the capacitance (E), and the input conductance (gin) (F) in the DA neurons with the A53T mutation compared to the control DA neurons.

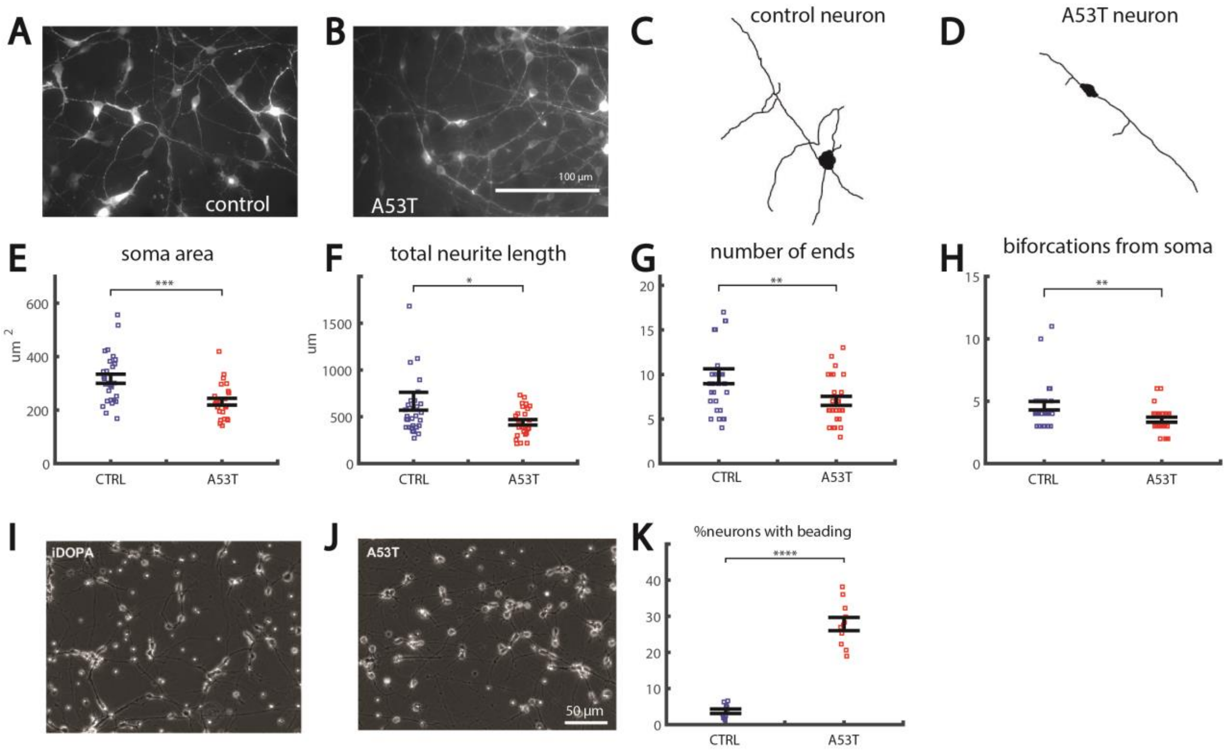

Supplementary Figure 7. A53T DA neurons are smaller and less arborized than control neurons. A. A representative image of a control dopaminergic neuronal culture. B. A representative image of an A53T dopaminergic neuronal culture. C. A representative trace of a control DA neuron. D. A representative traced A53T DA neuron. E. The soma area is reduced in A53T neurons. F. The total neurite length is reduced in A53T neurons. G. The total number of neurite ends is reduced in A53T neurons, indicating a less arborized neurite tree. H. The total number of neurites emerging out of the soma is reduced in A53T neurons. I-J. Bright-field images of representative beading in A53T cultures. K. The total number of neurites with beading is significantly larger in A53T cultures. * p value<0.05, **p value<0.01, ***p<0.001. Error bars represent the standard error in this figure.

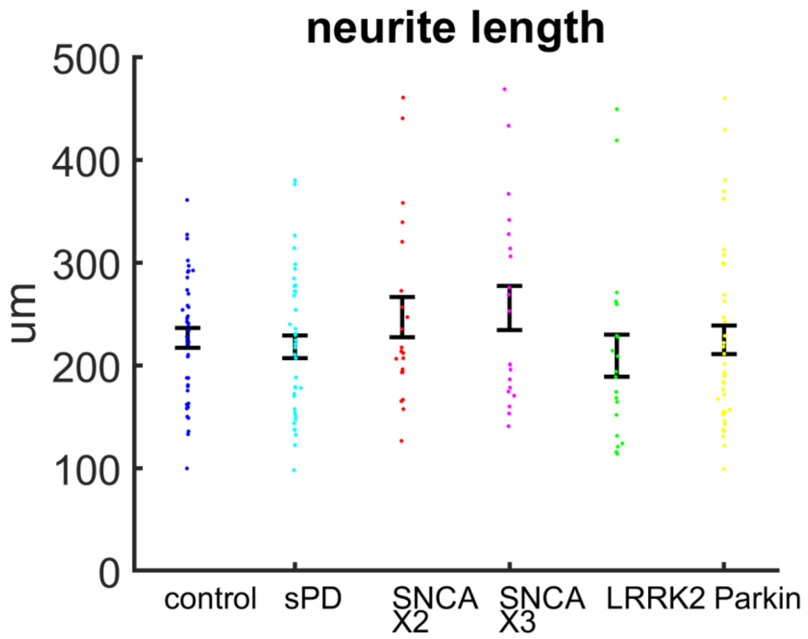

Supplementary Figure 8. The neurite length using the Kriks et al.[78] protocol for the midbrain neurons derived from the PD patients was measured after TH staining. No significant differences were observed between the different PD mutations, sporadic PD, and the controls.

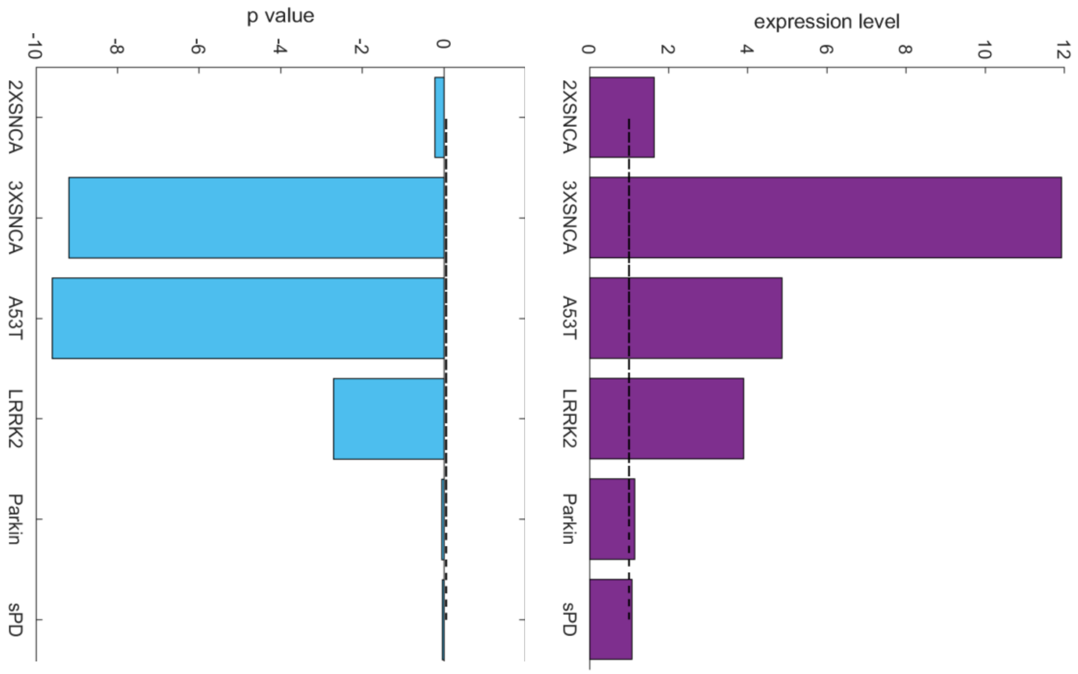

Supplementary Figure 9. The SNCA gene is dysregulated in some of the PD lines. A significant differential expression is observed in the following PD lines: LRRK2, A53T, and the SNCA triplication line.

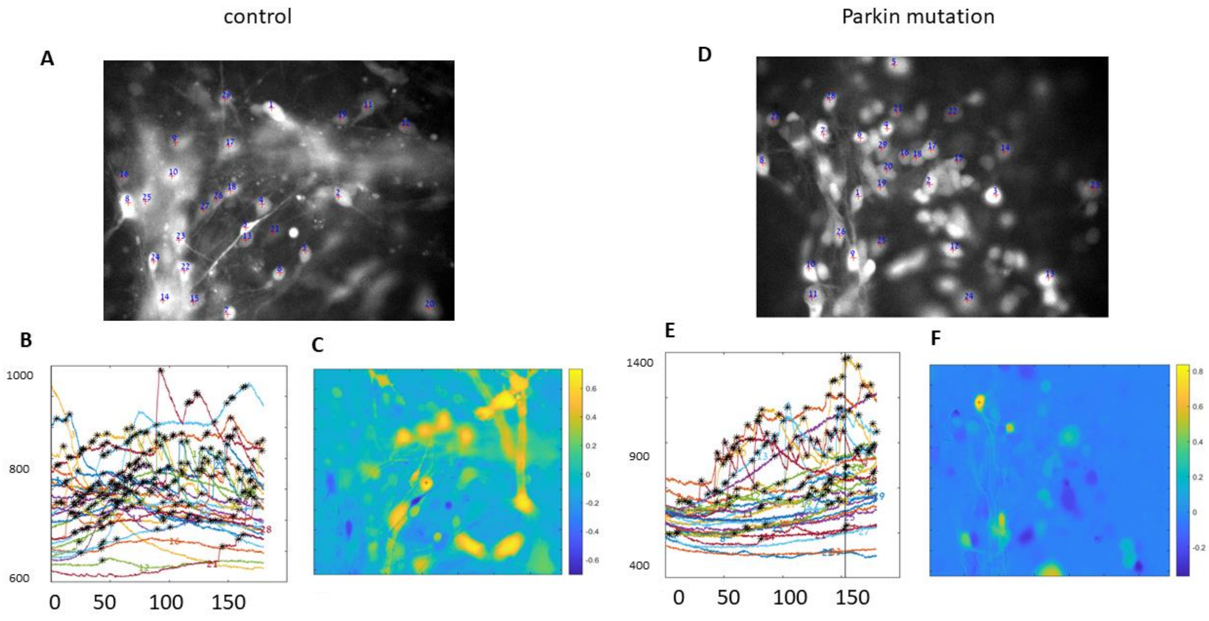

Supplementary Figure 10. Calcium imaging. A. Calcium imaging from a dopaminergic control neuronal culture. B. The fluorescent levels of the neurons marked in A. C. Correlations between control spiking neurons. D. Calcium imaging from a dopaminergic PD neuronal culture with the Parkin mutation. E. The fluorescent levels of the neurons marked in D. F. Correlations between spiking neurons derived from the PD patient with the Parkin mutation are much smaller compared to the control line, indicating a less connected network.

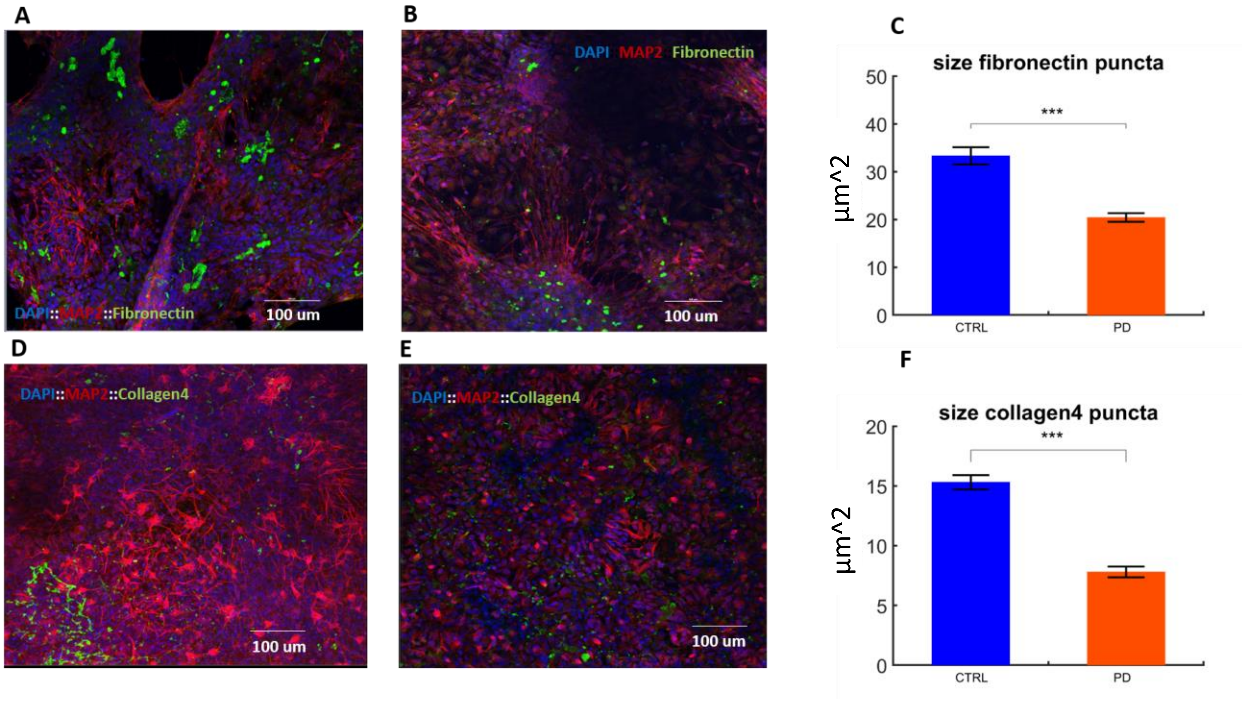

Supplementary Figure 11. Smaller Fibronectin and collagen 4 puncta. A. Example image of ICC of MAP2 and Fibronectin in a control dopaminergic neuronal culture. B. Similarly, an example image of ICC of MAP2 and Fibronectin in a sPD dopaminergic neuronal culture. The puncta appear smaller when compared to the control lines. C. Quantification of the size of the puncta over the following PD lines: LRRK2 mutation, sPD, and the SNCA triplication compared to two control lines. D. Example image of ICC of MAP2 and collagen4 in control lines. E. Similarly, example image of ICC of MAP2 and collagen4 in a sPD line. The collagen appears in longer fibers. F. Quantification of the collagen fiber puncta size over the following PD lines: LRRK2 mutation, sPD, and the SNCA triplication compared to two control lines. ***$p<0.001$. Error bars represent the standard error in this figure.

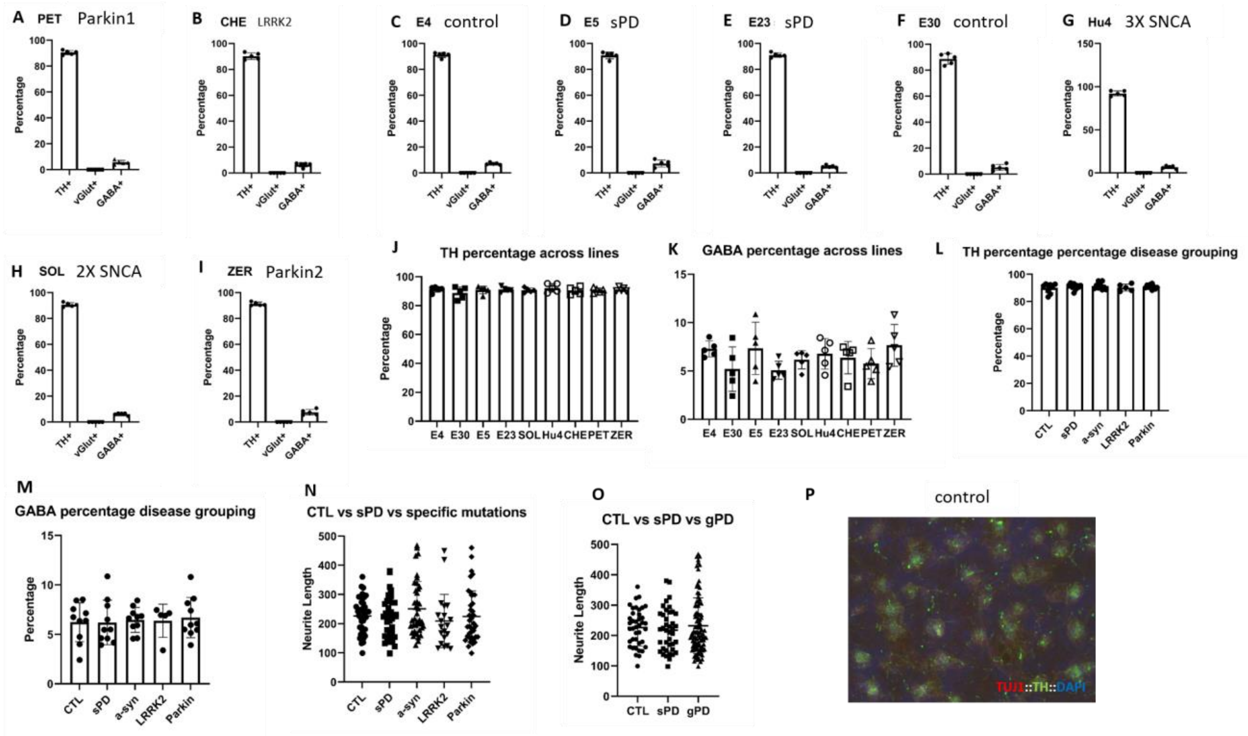

Supplementary Figure 12. Distribution of neuron types. Percentage of TH+/vGlut1+/GABA+ in the neuronal culture in the following lines derived from PD patients or healthy controls A. A Parkin mutation. B. A LRRK2 mutation. C. A control line. D. sPD. E. sPD. F. A control line. G. A triplication of the SNCA gene. H. a duplication of the SNCA gene. I. Another Parkin mutation. J. A summary of all lines for TH percentage. K. A summary of all lines for GABA percentage. L. TH percentage with a grouping of mutation types. M. GABA percentage with a grouping of mutation types. N. Neurites length for the different mutation types. O. Neurite lengths for control, sPD, and genetic PD. P. An example of Tuj1::TH::DAPI staining for a control line.

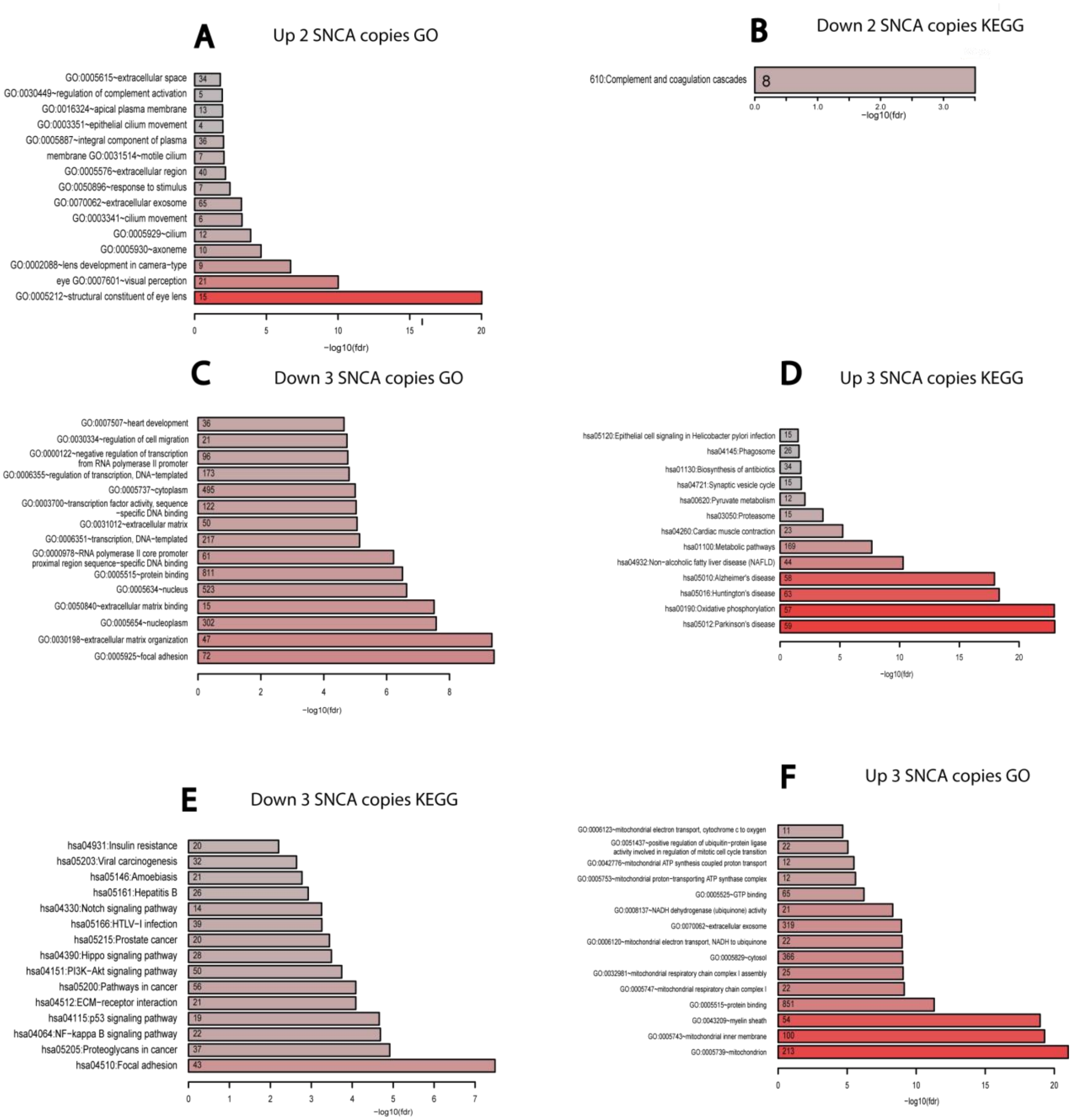

Supplementary Figure 13. Bar plots showing top overrepresented terms or pathways among differential gene sets. Numbers indicate counts of overlapping genes; red color represents the significance of the overlap. A. Up-regulated GO terms sets in the 2X patient compared to controls. B. Down-regulated KEGG gene set in the 2X patient compared to controls. C. Down-regulated GO terms in the 3X patient compared to controls. E. Up-regulated KEGG pathways in the 3X patient compared to controls. D. Down-regulated KEGG pathways in the 3X patient compared to controls. F. Up-regulated GO terms in the 3X patient compared to controls.

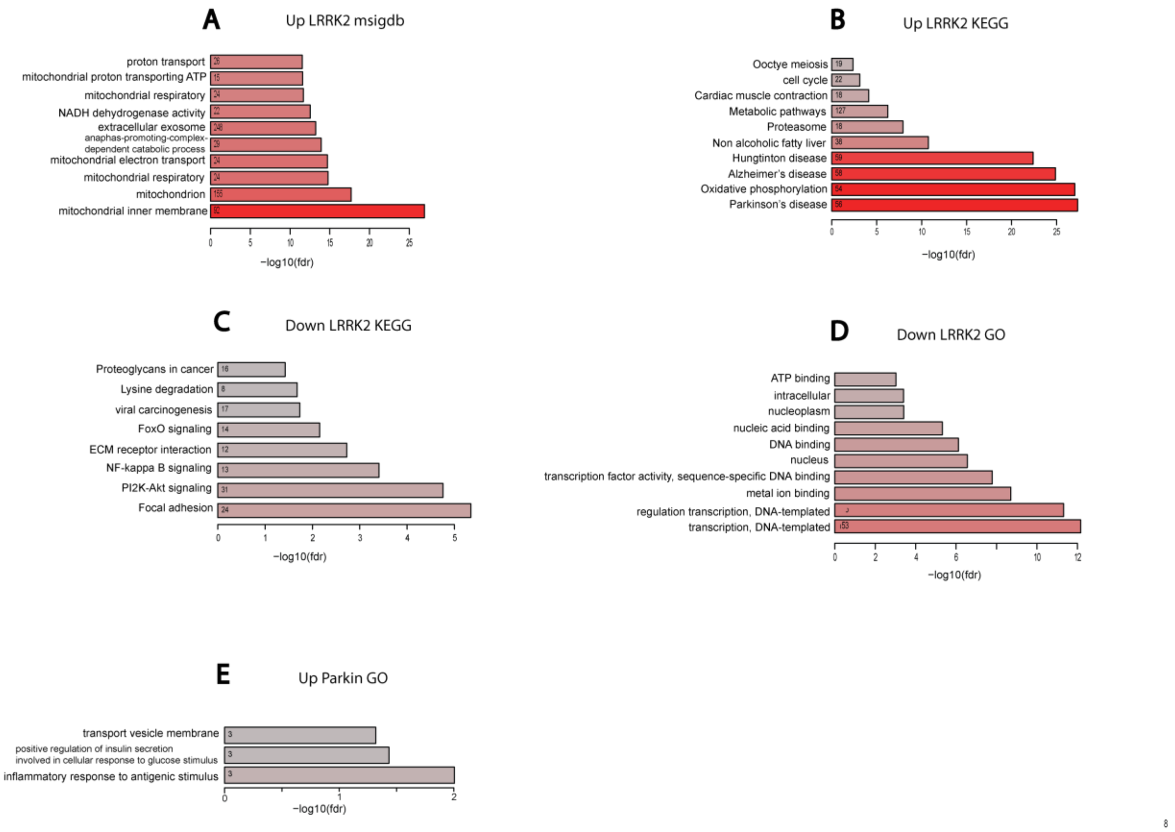

Supplementary Figure 14. Bar plots showing top overrepresented terms or pathways among differential gene sets. Numbers indicate counts of overlapping genes; red color represents the significance of the overlap. A. Up-regulated msigdb terms in the neurons of the LRRK2 patient compared to controls. B. Upregulated KEGG pathways in neurons of LRRK2 patient compared to controls. C. Down-regulated KEGG pathways in neurons of LRRK2 patient compared to controls. D. Down-regulated GO terms in the DA neurons of the Parkin patient compared to controls. E. Up-regulated GO terms in the DA neurons of the Parkin patient compared to controls.

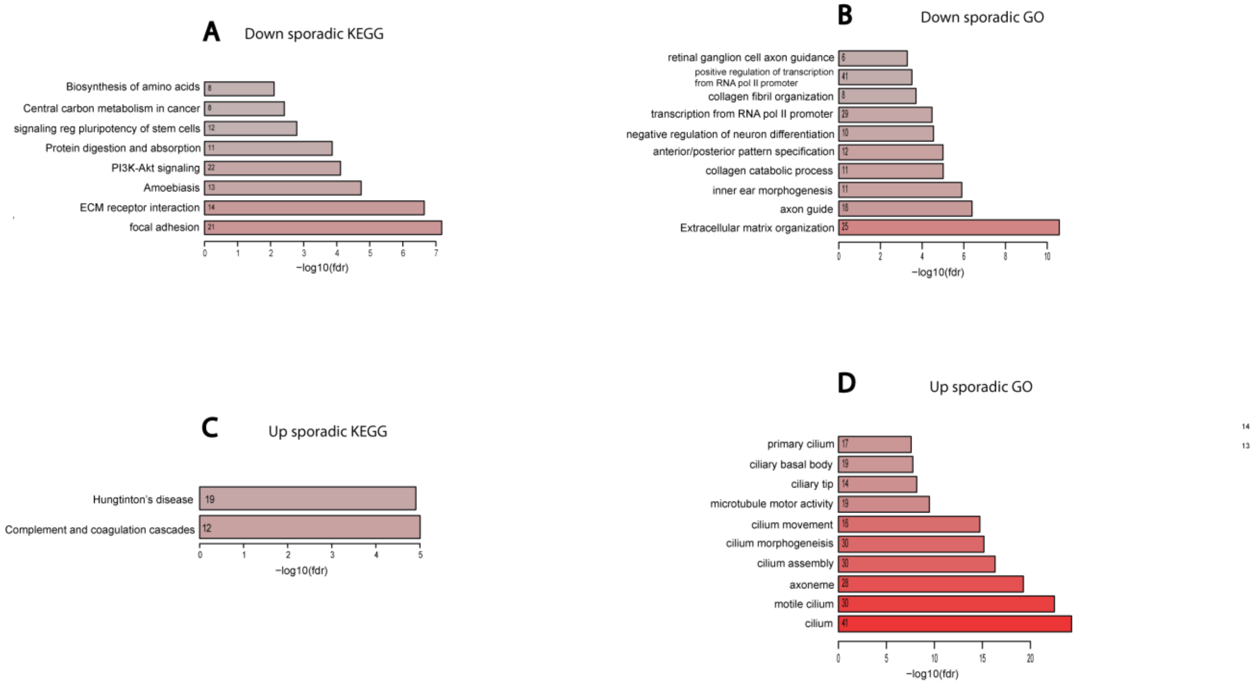

Supplementary Figure 15. Bar plots showing the top overrepresented terms or pathways among differential gene sets. Numbers indicate counts of overlapping genes; red color represents the significance of the overlap. A. Down-regulated KEGG pathways in neurons of sPD patients compared to controls. B. Down-regulated GO terms in the neurons of the sPD patients compared to controls. C. Up-regulated KEGG pathways in neurons of sPD patients compared to controls. D. Up-regulated GO terms in the neurons of the sPD patients compared to controls.

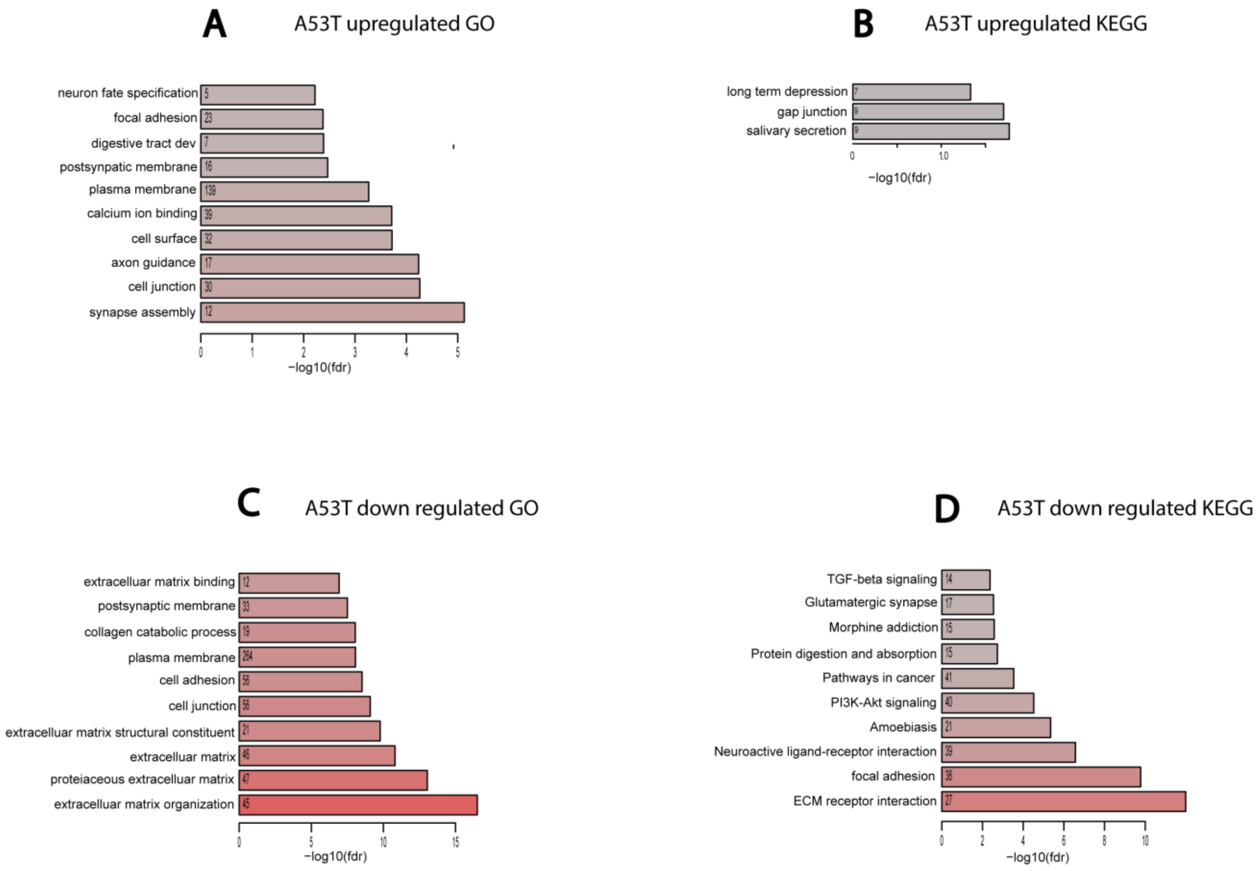

Supplementary Figure 16. Bar plots showing top overrepresented terms or pathways among differential gene sets. A. Up-regulated GO terms in the A53T neurons compared to control neurons. B. Up-regulated KEGG pathways in DA A53T neurons compared to control neurons. C. Down-regulated GO terms in the A53T neurons compared to control neurons. D. Down-regulated KEGG pathways in A53T neurons compared to control neurons.

Supplementary Table 1. A description of the first cohort of patients.

Patient	Affected	Sex	Age	comments
UKERfRJO-X-001	PD	M	45	heterozygous missense mutation in the EIF4G1 gene
UKERfR66-X-001	PD	M	55	
UKERfAY6-X-001	PD	M	37	
UKERfO3H-X-001	Control	M	71	
Hu.F4.17	a-Syn (triplication)	M	48	
UKERf1JF-X-001	Control	M	43	
UKERfM89-X-001	PD	M	65	
UKERf33Q-X-001	Control	F	45	

Supplementary Table 2. A description of the second cohort of patients.

Sample code	Family code	Mutation	Sex	Age at sampling
40102	12245	Control	M	53
38444	14605	LRRK2 HET G2019S	M	69
39840	14345	αSyn HET duplication	F	45
39720	8969	parkin ex2del/c.202-203delAG	M	65
39401	13738	parkin ex4del/c.255delA	F	45

Supplementary Table 3. A table summarizing all the GO terms and KEGGS significantly affected pathways in all the comparisons presented throughout the study.

Supplementary Table 4. A complete list of all the differentially expressed genes (DEGs) in the DA neurons derived from PD patients compared to controls.

Supplementary Table 5. A table summarizing all the MSigDB significantly affected pathways in all the comparisons presented throughout the study.

Supplementary Table 6. A table summarizing the differentially expressed genes in pathways that are related to synaptic activity.
